# Dose–response relationship of serum ferritin and dietary iron intake with metabolic syndrome and non-alcoholic fatty liver disease incidence: a systematic review and meta-analysis

**DOI:** 10.3389/fnut.2024.1437681

**Published:** 2024-10-01

**Authors:** Lu Yu, Ting Que, Yifeng Zhou, Zhengtao Liu

**Affiliations:** ^1^School of Medicine, Zhejiang Chinese Medical University, Hangzhou, China; ^2^Shulan International Medical College, Zhejiang Shuren University, Hangzhou, China; ^3^Key Laboratory of Artificial Organs and Computational Medicine in Zhejiang Province, Shulan International Medical College, Zhejiang Shuren University, Hangzhou, China; ^4^Shulan (Hangzhou) Hospital, Hangzhou, China; ^5^Birth Defects Prevention and Control Institute, Maternal and Child Health Hospital of Guangxi Zhuang Autonomous Region, Nanning, China; ^6^NHC Key Laboratory of Combined Multi-organ Transplantation, Key Laboratory of the Diagnosis and Treatment of Organ Transplantation, School of Medicine, Chinese Academy of Medical Sciences, First Affiliated Hospital, Zhejiang University, Hangzhou, China; ^7^Key Laboratory of Organ Transplantation, First Affiliated Hospital, School of Medicine, Zhejiang University, Hangzhou, China

**Keywords:** serum ferritin, dietary iron, metabolic syndrome, non-alcoholic fatty liver disease, meta-analysis, dose–response relationship

## Abstract

**Aim:**

This study aims to assess the dose–response impact of iron load on systemic and hepatic metabolic disorders including metabolic syndrome (MetS) and non-alcoholic fatty liver disease (NAFLD).

**Methods:**

Serum ferritin (SF) and dietary iron intake were selected to represent the indicators of iron load in the general population. PubMed, EMBASE and Web of Science databases were searched for epidemiological studies assessing the impact of SF/dietary iron intake on MetS/NAFLD occurrence. All literature was published before September 1st, 2023 with no language restrictions.

**Results:**

Fifteen and 11 papers were collected with a focus on connections between SF and MetS/NAFLD, respectively. Eight papers focusing on dietary iron and MetS were included in the following meta-analysis. For the impact of SF on MetS, the pooled odds ratio (OR) of MetS was 1.88 (95% CI: 1.58–2.24) for the highest versus lowest SF categories. In males, the OR was 1.15 (95% CI: 1.10–1.21) per incremental increase in SF of 50 μg/L, while for females, each 50 μg/L increase in SF was associated with a 1.50-fold higher risk of MetS (95% CI: 1.15–1.94). For connections between SF and NAFLD, we found higher SF levels were observed in NAFLD patients compared to the control group [standardized mean difference (SMD) 0.71; 95% CI: 0.27–1.15], NASH patients against control group (SMD1.05; 95% CI:0.44–1.66), NASH patients against the NAFLD group (SMD 0.6; 95% CI: 0.31–1.00), each 50 μg/L increase in SF was associated with a 1.08-fold higher risk of NAFLD (95% CI: 1.07–1.10). For the impact of dietary iron on MetS, Pooled OR of MetS was 1.34 (95% CI: 1.10–1.63) for the highest versus lowest dietary iron categories.

**Conclusion:**

Elevated SF levels is a linear relation between the incidence of MetS/NAFLD. In addition, there is a positive association between dietary iron intake and metabolic syndrome. The association between serum ferritin and metabolic syndrome may be confounded by body mass index and C-reactive protein levels.

## 1 Introduction

Metabolic syndrome (MetS) affects at least one-fourth of the global adult population (1, 2), presenting a complex array of clinical features including abdominal obesity [waist circumference (WC) >90 cm for men, WC > 80 cm for women], dyslipidemia [triglyceride (TG) >150 mg/dL], hyperglycemia [fasting blood glucose (FBG) ≥ 100 mg/dL or medication for anti-hyperglycemia], and hypertension [systolic blood pressure (SBP) ≥ 130 mmHg and/or diastolic blood pressure (DBP) ≥ 85 mmHg or the medication of anti-hypertension] (3). MetS surpasses its benign nature, significantly amplifying the risk of CVD and T2DM events by approximately two-and six-fold, respectively (4, 5).

Non-alcoholic fatty liver disease (NAFLD) stands as the most prevalent chronic liver ailment worldwide, with a prevalence of 25.24% among the general population (6). Often intertwined with insulin resistance (IR) and MetS, NAFLD commonly coexists with type 2 diabetes mellitus (T2DM), dyslipidemia, obesity, and hypertension (7).

Serum ferritin (SF), a ubiquitous intracellular protein pivotal in regulating iron homeostasis, serves as an established biomarker for assessing body iron stores (8). Nevertheless, mounting evidence suggests that elevated body iron stores may correlate with adverse health outcomes.

Iron is an essential trace element for the human body. Its sources can be divided into heme iron food sources, such as liver, animal blood, beef, lean meat, and fish, and non-heme iron food sources, such as spinach, beans, pumpkin seeds, and beetroots (9). It plays a crucial role in numerous cellular processes including signaling, respiration, DNA replication and synthesis, nucleic acid repair, and energy metabolism (10). The consumption, uptake, transfer, and storage of iron are important for maintaining iron balance in the body (11). However, excessive levels of iron can lead to inflammation and tissue damage by generating harmful hydroxyl radicals through oxidative reactions. These radicals cause oxidative damage to important cellular components like lipids, proteins, and DNA (12). Considering that oxidative stress and inflammation contribute significantly to the development of Metabolic Syndrome (MetS), there exists a close association between dietary iron levels and MetS (13).

Considering the close association between markers of iron metabolism and metabolic derangements, numerous studies have investigated the relationships between SF/dietary iron and the occurrence of MetS/NAFLD. Moreover, past studies have explored the effects of SF/dietary iron on metabolic diseases (14–19). However, inconsistent and controversial findings suggest that potential confounding factors may influence the predictive role of SF/dietary iron in monitoring MetS and NAFLD. We have experience on quantitative evaluation of risk covariates on specific disease (20–22). Therefore, we conducted a systematic review and meta-analysis to quantitatively assess the trend of MetS/NAFLD incidence associated with SF/dietary iron variations based on published literature (23–56). Subgroup analysis was employed to explore latent confounders. Therefore, we conducted a systematic review and meta-analysis to quantitatively assess the trend of MetS/NAFLD incidence associated with SF/dietary iron variations based on published literature (23–56). Subgroup analysis was employed to explore latent confounders. Comprehensive dose–response analyses are warranted to elucidate the effects of quantitative SF/dietary iron variables on the persistent risk of metabolic diseases.

## 2 Materials and methods

### 2.1 Search strategy

We conducted a systematic evaluation and meta-analysis following the guidelines of Preferred Reporting Entries for Systematic Evaluation and Meta-Analysis (PRISMA) (refer to Supplementary material). A thorough search was performed in Medline, Embase, and Web of Science (WOS) databases from the time of establishment until February 10, 2023 (without any language limitations). We utilized various terms such as “Metabolic syndrome,” “MetS,” “Non-alcoholic fatty liver disease,” “non-alcoholic Steatohepatitis,” “NAFLD,” “NASH,” “Iron,” “Fe,” “Serum ferritin” and “SF” to retrieve relevant literature. In case any pertinent studies were missed during the initial search, we also manually retrieved additional references. The detailed database search strategy can be found in Supplementary Table S1.

### 2.2 Study selection

The study inclusion criteria for this meta-analysis were (1) observational studies examining the association between SF/iron levels and MetS/NAFLD; (2) studies that directly provided effect sizes (corrected for age, gender, smoking, alcohol consumption, family history, hs-CRP, BMI, etc.), including risk ratios (HR), odds ratios (OR), standardized mean differences (SMD), 95% confidence intervals (CI), or had enough data to calculate these metrics; (3) studies published in English. Studies that met the following exclusion criteria were not included in the meta-analysis: (1) reviews or other types of non-original studies; (2) non-breeding trials; (3) studies that used pregnant women as the study population; (4) had other complications that could affect SF/iron detection; (5) incomplete information or inability to obtain the necessary information by contacting the corresponding authors; and (6) duplicate study populations. In the case of multiple studies on the same population, only one data set containing more individuals or more comprehensive data was included, depending on the specific analytic needs.

### 2.3 Quality assessment

Two investigators independently assessed the quality of each study, and any disagreements were resolved by a third author. The included studies were cross-sectional and were evaluated using the American Association for Healthcare Research and Quality (AHRQ) scale (57). The evaluation tool includes 11 entries for the recommended criteria. A score of 1 was assigned to “yes” and 0 to “no” or “unclear.” 0 to 3 was categorized as a low-quality study, 4–7 as a moderate-quality study, and 8–11 as a high-quality study.

### 2.4 Data extraction

Two investigators (LY and YZ) independently collected data from specific eligible articles. The information recorded included study design (prospective cohort, cross-sectional, and other designs), association metrics used (ratio or risk ratio), country of origin, population, sex, mean participant age, number of participants, ferritin assay technique, ferritin levels, metabolic syndrome criteria and outcomes, histologic extent of NAFLD (if any), methods of NAFLD assessment, and additional information (categories of NAFLD, casel/control number).

### 2.5 Statistical analysis

Initially, we combined the results of the included studies using a random-effects model (inverse variance) of selected ratio ratios (for cross-sectional studies) and standardized mean differences (for prospective cohort studies), as well as 95% confidence intervals, to quantify the associations between SF and MetS, dietary iron and MetS, and SF and NAFLD. If significant heterogeneity existed, a random effects model was used and heterogeneity was assessed using the *I*^2^ statistic (low, high, and medium heterogeneity defined as 25, 50, and 75%, respectively) (58). First, a meta-analysis was conducted on the highest quartile/third quartile/quartile/quintile versus the lowest or reference quartile. Heterogeneity between studies was then assessed using the *I*^2^ statistic. If no statistically significant heterogeneity was found (*I*^2^ < 50% and *p* > 0.10), the Mantel–Haenszel fixed-effects model would be applied (59). Otherwise, the DerSimonian-Laird random effects model will be used (60). We performed subgroup analyses to explore potential sources of variation, such as geographic region (East-Asian and Non-East-Asian), diagnostic criteria for metabolic syndrome [International Diabetes Foundation (IDF), Adult Treatment Panel (ATP) III, and Others], ferritin assay (chemiluminescence, immunoradiometric, immunoturbidimetric, not reported), study design (case–control, and cross-sectional and prospective study) and so on. Sensitivity analyses were performed using a systematic exclusion of 1 study at a time to assess its effect on the overall effect size.

We used the method developed by Greenland and Longnecker (61), which extracts the following data from studies that provide information on at least 3 quantitative exposure categories: SF/dietary level, number of cases and participants, OR, and 95% CI. Based on the data obtained, the median or mean SF level was assigned to each category. Where median or mean values were not provided for each category, we used the midpoint estimate between the upper and lower bounds. If the highest category had an open range, we assumed it was the same width as the second-highest category (62). To assess potential linear or nonlinear associations between SF and MetS, Dietary iron and MetS, and SF and NAFLD, we used restricted cubic spline with four nodes located at the 5, 35, 65, and 95% fixed percentiles of the distribution. In the absence of nonlinear associations observed in the dose–response analyses, we performed a meta-analysis focusing exclusively on linear trends to examine the association between each 50 μg/L increase in SF and the risk of MetS/NAFLD across sexes. Egger’s test and Begg’s test (63) were used to assess publication bias. Stata 14.0 software was used for statistical analysis. A *p* < 0.05 was considered statistically significant unless explicitly stated otherwise. Our study flow diagram was shown in Supplementary Figure S1.

## 3 Results

### 3.1 Literature retrieval

**Figure 1:**
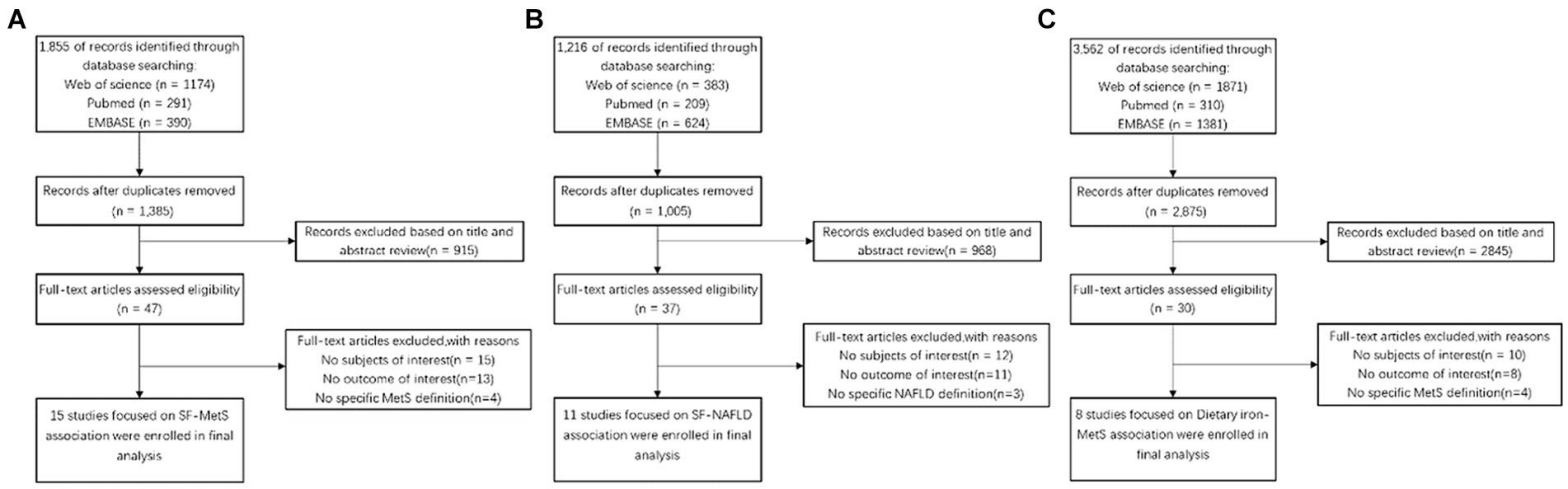
Flow diagram of eligible literature selection. **(A)** Literature on the association between SF and MetS; **(B)** literature on the association between SF and NAFLD; **(C)** literature on the association between Dietary and MetS. SF, serum ferritin; MetS, metabolic syndrome; NAFLD, non-alcoholic fatty liver disease.

We conducted a comprehensive screening of 5,067 publications that may be relevant to this study. After removing 1,566 duplicates from three databases (PubMed, Embase, and WOS), we finalized 34 eligible articles. These included 15 articles on the relationship between SF-MetS, 11 articles on SF-NAFLD, and eight articles on dietary iron-MetS. Inter-rater agreement was high (Cohen’s kappa = 0.766). See Figure 1 for a flowchart of our literature selection process.

### 3.2 Quality assessment, study characteristics, and bias assessment

All included studies were of high quality according to the AHRQ evaluation system. The AHRQ scores ranged from 7 to 11 with a mean of 8.17 points. For more details on the quality assessment, see Supplementary Table S3.

Table 1 summarizes the characteristics of the included studies. In our meta-analysis, we analyzed data from 15 cross-sectional studies (23–37) examining the relationship between SF and MetS. These studies involved a total sample size of 89,205 individuals and were conducted before 2003 in China, Korea, the United States, Croatia, Europe, and Spain, with participants aged between 18 and 80 years.

**Table 1:** Characteristics of the thirty-four studies included in meta-analysis.

**First author, publication year [ref]**	**Country**	**Geographic area**	**Study design**	**Sample(female/male)**	**Age years**	**Ferritin assay**	**Mets criteria/ NAFLD diagnosis approach/tool**	**Out-come**	**OR/RR**	**Calculation** **method**	**Adjusted covariates**	**Categories of NAFLD [Male/Female, mean age (years)]NAFL/NASH**	**Casel/Control Number [Male/Female, mean age (years)]**
Wang et al., 2020 (37)	China	Asian	Cross-sectional	1659 (1098/651)	50.16±17.42	CLIA	ATP III	SF-MetS	Male OR:1.05 (0.53, 2.07) Female OR:0.91 (0.51, 1.64)	Logistic regression models	Age and BMI	NR	NR
Li et al., 2013 (31)	China	Asian	Cross-sectional	8441 (4502/3939)	51.1 (24.5-75.9)	RIA	ATP III	SF-MetS	Male OR: 4.05 (3.19, 5.14) Female OR: 2.34 (1.84, 2.97)	Multivariable logistic regression analysis	Age, nationality, smoking status, alcohol consumption and hs-CRP in different models	NR	NR
Chen et al., 2017 (24)	China	Asian	Cross-sectional	2786 (1462/1324)	25–74	CLIA	IDF	SF-MetS	Male OR: 1.77 (1.23, 2.56) Female OR: 1.67 (1.09, 2.56)	Multiple linear regression models	Age, serum creatinine, alanine aminotransferase, neutrophil/lymphocyte ratio, hemoglobin, HOMA-IR, and frequency of pork consumption	NR	NR
Jehn et al., 2004 (26)	America	American	Cross-sectional	5949 (3069/2880)	> 20	RIA	ATP III	SF-MetS	Male OR:1.6 (0.9, 2.7) premenopausal women OR: 2.4 (1.1, 5.2) postmenopausal women OR: 2.7 (1.7, 4.1)	Logistic regression models	Age, race/ethnicity, CRP, smoking, alcohol intake, and BMI.	NR	NR
Suárez-Ortegón et al., 2016 (34)	Croatia	Europe	Cross-sectional	725 (441/284)	≥ 18	CLIA	ATP III	SF-MetS	Male OR: 2.02 (1.51, 2.70) premenopausal women OR: 1.24 (0.85, 1.80) postmenopausal women OR: 1.65 (1.11, 2.46)	Multiple linear regression models	Age, fibrinogen levels, smoking status and alcohol consumption	NR	NR
Shim et al., 2017 (33)	Korea	Asian	Cross-sectional	15963 (9006/6957)	16–80	RIA	IDF	SF-MetS	Male OR:1.65 (1.28, 2.12) Female OR: 1.36 (1.09, 1.69)	Multiple linear regression models	Age, body mass index, white blood cell, hypertension, dyslipidemia, cerebrovascular disease, coronary heart disease, diabetes, residence area, smoking, alcohol intake, physical activity, education level, total intake, total energy intake, protein intake, fat intake, and carbohydrate intake	NR	NR
Kim et al., 2011 (28)	South Korea	Asian	Cross-sectional	12090 (5712/6378)	20-89	TIA	NCEP ATPIII	SF-MetS	Male OR: 1.58 (1.06, 2.37) Female OR: 1.07 (0.71, 1.63)	Multivariable logistic regression analysis	Age, BMI, hsCRP, smoking, alcohol use, menopause status in women, AST, ALT, and GGT	NR	NR
Lee et al., 2011 (30)	South Korea	Asian	Cross-sectional	6311 (3627/2684)	36-63	RIA	NCEP-ATP III	SF-MetS	Male OR: 1.80 (1.09, 2.97) premenopausal women OR: 3.57 (1.38, 9.21) postmenopausal women OR: 1.54 (0.90, 2.65)	Logistic regression.	Age, educational level, smoking, alcohol intake, BMI, AST, and ALT.	NR	NR
Kang et al., 2012 (27)	Korea	Asian	Cross-sectional	7346 (4114/3232)	48	RIA	NCEP ATPIII	SF-MetS	OR: 1.22 (0.91, 1.64)	Multivariable logistic regression analysis	Age, BMI, WBC count, alcohol consumption, smoking status, daily energy intake, menopausal status, HOMA-IR	NR	NR
Chang et al., 2013 (23)	Taiwan	Asian	Cross-sectional	2654 (1513/1141)	55	EIA	NCEP ATPIII for Asia Pacific.	SF-MetS	OR: 1.72(1.21, 2.45)	Multivariable logistic regression analysis	Age, sex, BMI, inflammation (GOT, GTP, ALK, amylase, BUN, UA, creatinine, homocysteine), lifestyle factors (past smoker, drinking habits and betel nut consumpfion), iron status (hemoglobin, IDA) and self-reported family health history of chronic diseases (hyperlipidemia, fatty liver disease, hypertension, diabetes mellitus)	NR	NR
Cho et al., 2011 (25)	Korea	Asian	Cross-sectional	3082 (3082/0)	41	CLIA	ATP III	SF-MetS	Premenopausal women OR:1.21 (0.71, 2.08) Postmenopausal women OR: 1.62 (1.04, 2.51)	Multivariable logistic regression analysis	Age; body mass index; alcohol intake; smoking history; exercise; hormone therapy use; hemoglobin, aspartate aminotransferase, and alanine aminotransferase levels; and intake of energy and iron	NR	NR
Ryoo et al., 2011 (32)	Korea	Asian	Cross-sectional	18581 (0/18581)	41	CLIA	ATP III	SF-MetS	OR: 1.99 (1.70–2.33)	Logistic regression models	Age, alcohol intake, recent smoking status, total protein, GGT, log(hsCRP), WBC, ALT, ApoB, TIBC, serum creatinine and HOMA-IR.	NR	NR
Ledesma et al., 2015 (29)	Spanish	Europe	Cross-sectional	3386 (0/3386)	19-65	TIA	ATP III	SF-MetS	OR: 1.92 (1.48 –2.49)	Multivariable logistic regression analysis	Age, history of blood donation, transaminases, and alcohol intake	NR	NR
Tang et al., 2015 (36)	China	Asian	Cross-sectional	2417 (0/2417)	20–73	CLIA	ATP III	SF-MetS	OR: 2.29 (1.47, 3.54)	Binary logistic regression model	Age, physical activity, family history of chronic diseases (4 covariates: hypertension, diabetes mellitus, stoke, coronary heart disease), lifestyle factor of alcohol drinking status and smoking status. BMI.	NR	NR
Chandok et al., 2012 (38)	Canada	Europe	Prospective study	NAFL: 60 (37/23) NASH: 28 (13/15)Case: 88 Control: NA	>18	NA	Liver biopsy	SF-NAFLD	NR	Multivariable logistic regression analysis	NR	NAFL: 60 (37/23) NASH: 28 (13/15)	Case: 88 Control: NA
Hotta et al., 2010 (41)	Japan	Asian	Case-control	NAFL: 64 (23/41, 51) NASH: 189 (99/90, 51) Case: 253 (122/131, 51) Control: 578 (182/396, 47)	>18	NA	Liver biopsy	SF-NAFLD	NR	Multivariable logistic regression analysis	NR	NAFL: 64 (23/41, 51) NASH: 189 (99/90, 51)	Case: 253 (122/131, 51) Control: 578 (182/396, 47)
Hsiao et al., 2004 (42)	Taiwan, China	Asian	Case-control	Case: 43 (20/23, 33) Control: 167 (27/140, 36)	18-61	NA	Ultrasonography	SF-NAFLD	NR	Multivariable logistic regression analysis	NR	NA	Case: 43 (20/23, 33) Control: 167 (27/140, 36)
Goh et al., 2016 (40)	USA	American	Prospective study	NAFL: 114 (52/62,46) NASH: 291 (127/164, 49) Case: 405 (179/226, 48) Control: NA	>18	NA	Liver biopsy	SF-NAFLD	NR	Multivariable logistic regression analysis	NR	NAFL: 114 (52/62, 46) NASH: 291 (127/164, 49)	Case: 405 (179/226, 48) Control: NA
Rosa et al., 2013 (39)	Italy	Europe	Case-control	NAFL: 90 (43/47, 49) NASH: 110 (49/61, 53)Case: 200 (92/108) Control: 100 (48/52, 54.2)	45-67	NA	Liver biopsy	SF-NAFLD	NR	Multivariable logistic regression analysis	NR	NAFL: 90 (43/47, 49) NASH: 110 (49/61, 53)	Case: 200 (92/108) Control: 100 (48/52, 54.2)
Sazci et al., 2008 (45)	Turkish	Asian	Case-control	NASH: 57 (31/26,44)Case: 57 (31/26,44) Control: 245 (106/139, 45)	18-66	NA	Liver biopsy	SF-NAFLD	NR	Binary logistic regression model	NR	NASH: 57 (31/26, 44)	Case: 57 (31/26, 44) Control: 245 (106/139, 45)
Jiang et al., 2014 (43)	China	Asian	Cross-sectional	Case: 446 (351/95, 46) Control: 531 (201/330, 42)	20-60	NA	Liver biopsy	SF-NAFLD	NR	Logistic regression analysis	NR	NA	Case: 446 (351/95, 46) Control: 531 (201/330, 42)
Yoneda et al., 2010 (48)	Japan	Asian	Case-control	NAFL: 24 (48) NASH: 62 (52) Case: 86 Control: 20	>18	NA	Liver biopsy	SF-NAFLD	NR	Linear regression analysis	NR	NAFL: 24 (48) NASH: 62 (52)	Case: 86 Control: 20
Kim et al., 2018 (44)	Korea	Asian	Cross-sectional	Case: 100(59) Control: 141(58)	>18	NA	Ultrasonography	SF-NAFLD	NR	Multivariable logistic regression analysis	NR	NA	Case: 100 (59) Control: 141(58)
Yang et al., 2022 (64)	China	Asian	Cross-sectional	Case: 1604(856/748, 53) Control: 2085(950/1135, 45)	>18	NA	Liver biopsy	SF-NAFLD	NR	Multivariable logistic regression analysis	NR	NA	Case: 1604(856/748, 53) Control: 2085(950/1135, 45)
Tsuchiya et al., 2010 (46)	Japan	Asian	Case-control	NAFL: 17 (46) NASH: 11 (50) Case: 28 Control: 8(30)	>18	NA	Liver biopsy	SF-NAFLD	NR	Multivariable logistic regression analysis	NR	NAFL: 17 (46) NASH: 11 (50)	Case: 28 Control: 8 (30)
Azadbakh et al., 2009 (49)	Iran	Asian	Cross-sectional	482	40-60	24 h recall	NCEP ATP III	Dietary iron-Mets	OR: 2.13 (1.15, 3.97)	Multivariable logistic regression analysis	Age, physical activity, total energy intake, current estrogen use, menopausal status, family history of diabetes orstroke, intakes of dietary fiber and cholesterol, percent of energy from fat, fruit, and vegetables, white meats and fish, dairy, partially hydrogenated and nonhydrogenated vegetable oils, and whole- and refined-grains.	NR	NR
Bruscato et al., 2010 (50)	Brazil	SouthAmerican	Cross-sectional	284	>60	24 h recall	IDF	Dietary iron-Mets	OR: 1.72 (0.30, 1.72)	Multivariable logistic regression analysis	Age, smoking, years of education, physical activity and dietary fiber.	NR	NR
Otto et al., 2012 (51)	US	American	Cohort	3,828	45–84	24 h recall	AHA	Dietary iron-Mets	Heme iron OR: 1.06 (0.81, 1.40) Nonheme iron OR: 0.95 (0.70, 1.29)	Cox proportional hazard regression models	Energy intake, age, sex, race-ethnicity, education, study center, alcohol intake, physical activity, BMI, fiber intake, cigarette smoking, dietary supplement use, the ratio of polyunsaturated fat intake: saturated fat intake and antioxidant intake	NR	NR
Motamed et al., 2013 (54)	Iran	Asian	Cross-sectional	3,800	35–65	24 h recall	IDF	Dietary iron-Mets	OR: 1.12 (0.9, 1.4)	Logistic regression models	Sex, age, physical activity level, smoking, past medical history, energy intake and BMI	NR	NR
Zhu et al., 2018 (56)	China	Asian	Cross-sectional	3099 (1609/1430)	>18	24 h and 3 days recall	NCEP ATP III	Dietary iron-Mets	Total Iron OR: 1.59 (1.15, 2.20) Heme iron OR: 1.06 (0.80, 1.39) Nonheme iron OR: 1.44 (1.04, 1.99)	Multivariate generalized linear mixed models	Age, sex, income, physical activity level, intentional physical exercise, smoking status, alcohol use and dietary total energy intake	NR	NR
Vieira et al., 2018 (52)	Brazil	South Amerisan	Cross-sectional	591	>18	24 h recall	NCEP ATP III	Dietary iron-Mets	Total Iron OR: 1.14 (0.54–2.40) Heme iron OR: 2.39 (1.10–5.21) Nonheme iron OR: 1.05 (0.44–2.48)	Linear regression model	Physical activity, gender, alcohol consumption, household per capita income, BMI, high-sensitivity C-reactive protein, age, smoking status, race, total energy intake, misreporting, saturated fat and vitamin Cintakes	NR	NR
Esfandiar et al., 2019 (52)	Iran	Asian	Cohort	4,654	>18	24 h recall	NCEP ATP III	Dietary iron-Mets	Total Iron OR: 2.04 (0.97–4.28) Heme iron OR: 0.87 (0.67–1.12) Nonheme iron OR: 1.15 (0.80–1.63)	Multivariable Cox proportional hazard regression models	Age, sex, baseline BMI, educational level, smoking status, total energy intake, fiber, saturated fat, sodium, vitamin C and magnesium intakes	NR	NR
Zhu et al., 2020 (55)	China	Asian	Cross-sectional	5,323	>18	24 h and 3 days recall	NCEP ATP III	Dietary iron-Mets	Total Iron OR: 1.60 (1.21, 2.11) Heme iron OR: 0.78 (0.63, 0.96) Nonheme iron OR: 1.53 (1.16, 2.02)	Hierarchical logistic regression models	Age, sex, region, years of education, physical activity level, intended physical exercises, smoking status, alcohol use and daily energy intake, zinc and magnesium	NR	NR

Abbreviatins: ALK, Alkaline Phosphatase; ALT, Alanine Aminotransferase; AST, Aspartate Aminotransferase; ATP III, National Cholesterol Education Program Adult Treatment Panel III; AHA, American Heart Association; BMI, Body Mass Index; BUN, Blood Urea Nitrogen; CLIA, chemiluminescence immunoassay; GGT, Gamma-Glutamyltransferase; GOT, Glutamic-Oxaloacetic Transaminas; HOMA-IR, Homeostasis Model Assessment of Insulin Resistance; Hs-CRP, High Sensitivity C-Reactive Protein; IDA, Iron Deficiency Anemia; IDF, International Diabetes Federation; TIBC, Total Iron Binding Capacity; MetS, metabolic syndrome; NAFLD, nonalcoholic fatty liver disease; NA, not available; NR, not referred; RIA, immunoradiometric assay; SF, serum ferritin; TIA, immunoturbidimetric assay; UA, Uric Acid; WBC, White Blood Cell count.

Regarding the diagnostic criteria for MetS: eight studies used the International Diabetes Federation (ATPIII) guidelines; four referenced the modified National Cholesterol Education Program Adult Treatment Panel III (NCEP ATP III) guidelines; and two referenced the International Diabetes Federation (IDF) guidelines. In these studies, different methods were used to determine SF levels: chemiluminescence in six cases; immunoradiation in five cases; immunoturbidimetry in three cases; and electrochemiluminescence immunoassay in one case. For statistical analysis models: 11 results were calculated using multifactorial logistic regression models; three using multiple linear regression models; and one group using binary logistic regression models.

A total of 11 articles (38–46, 48, 64) were included in this study, focusing on the relationship between SF and NAFLD. Among these articles, two were prospective cohort studies, six were case-sectional studies, and three were cross-sectional studies. The diagnosis of NAFLD (NASH) was confirmed using hepatic ultrasonography in two studies and liver biopsy in nine studies. Geographically, two studies were conducted in Europe, eight in Asia, and one in America. The cumulative number of participants across all studies reached a substantial count of 8,302 individuals.

Eight articles (49–56) were selected to investigate the association between dietary iron intake and MetS. The diagnostic criteria for MetS varied among the included articles; five used NCEP ATP III criteria while two employed IDF criteria and one utilized American Heart Association (AHA) criteria. Out of these eight articles, six adopted a cross-sectional design while the remaining two followed a cohort design approach. Dietary iron levels were assessed through either 24-h or 3-day recall methods across all included studies with sample sizes ranging from 284 to 5,322 individuals resulting in a combined total sample size of 22,061 participants. Age, gender, and MetS components were corrected as key covariates in all studies.

### 3.3 SF and MetS incidence

#### 3.3.1 High versus low

**Figure 2:**
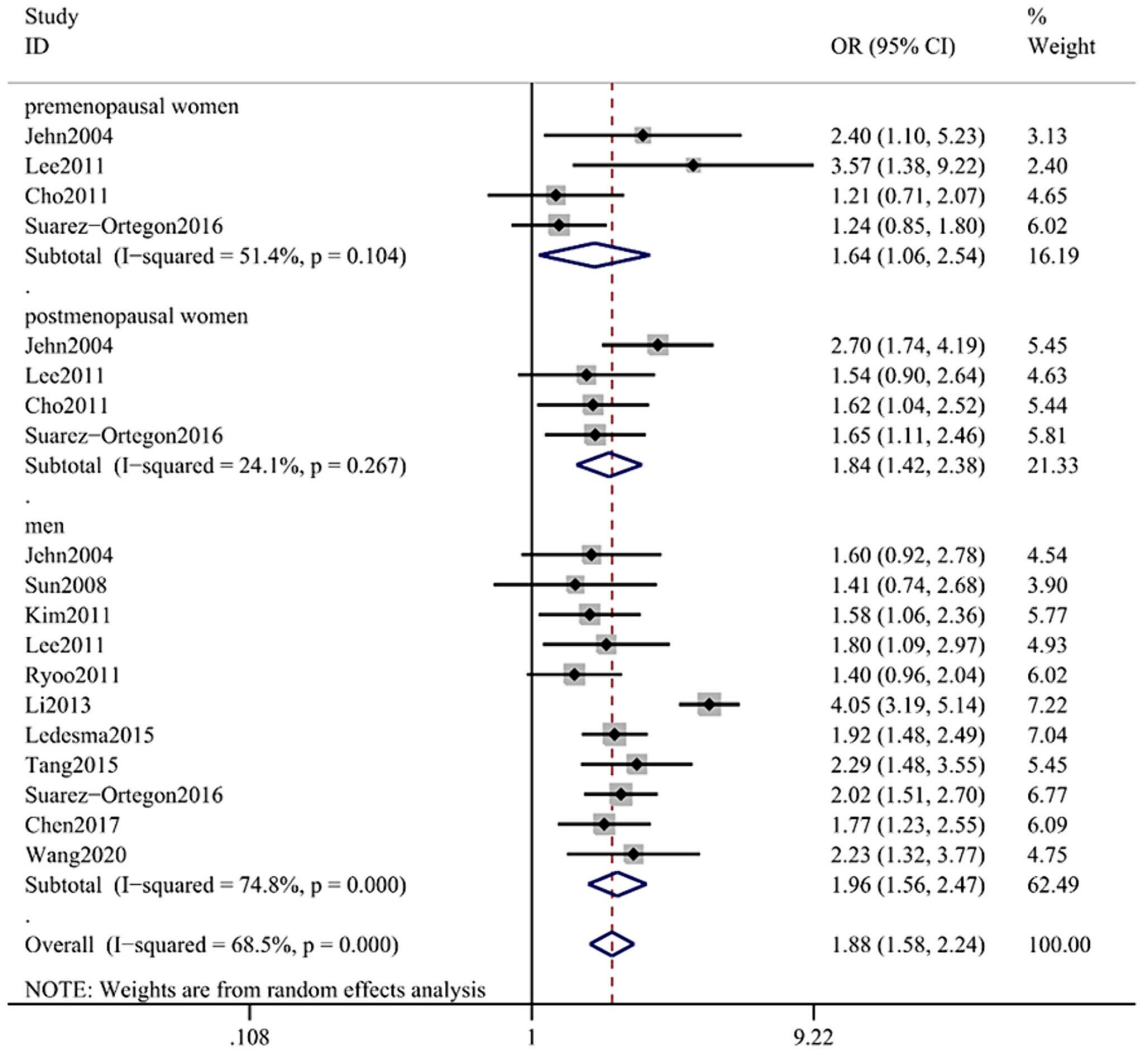
Forest plot of association between SF and MetS in cross-sectional studies. Pooled odds ratios of MetS compared between highest and lowest SF categories. SF, serum ferritin; MetS, metabolic syndrome.

The combined OR of the 15 included papers was 1.88 (95% CI: 1.58–2.44) with low heterogeneity (*p* < 0.001; *I*^2^ = 68.5%; Figure 2A). The pooled OR for men was 1.96 (95% CI: 1.56, 2.47); heterogeneity: *p* < 0.001; *I*^2^ = 74.8%; for premenopausal women it was 1.64 (95% CI: 1.06, 2.54); heterogeneity: *p* = 0.104; *I*^2^ = 51.4% and for postmenopausal women it was 1.84 (95% CI: 1.42, 2.38); heterogeneity: *p* = 0.267; *I*^2^ = 24.1%.

#### 3.3.2 Dose–response analysis

**Figure 3:**
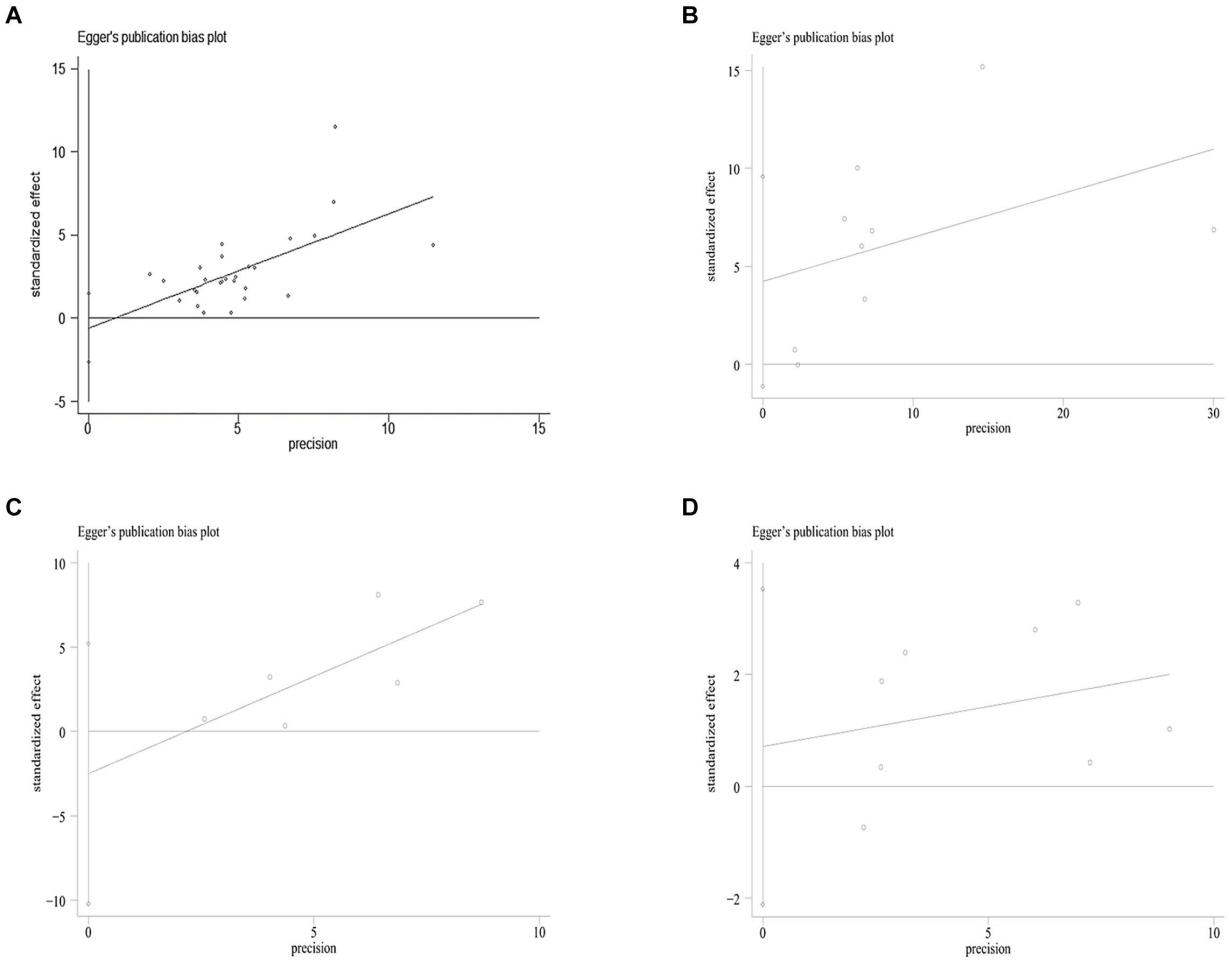
Egger’s funnel plot analysis of publication bias **(A)** between SF and MetS, Egger’s test: *p* = 0.561. **(B)** Between NASH/NAFLD and control, Egger’s test: *p* = 0.104. **(C)** Between NASH *VS* NAFLD, Egger’s test: *p* = 0.417. **(D)** Between Dietary Iron and MetS, Egger’s test: *p* = 0.560. SF, serum ferritin; MetS, metabolic syndrome; NAFLD, non-alcoholic fatty liver disease; NASH, non-alcoholic steatohepatitis.

Due to the limited literature on dose–response meta-analysis, only four papers were included (24, 26, 30, 31). A linear dose–response relationship between SF and MetS across genders is shown in Figures 3A,B, with a linear dose–response relationship between the two populations (male: *p* < 0.05, *P*-nonlinearity = 0.0516, female: *p* < 0.05, P-nonlinearity = 0.9269).

For every 50 μg/L increase in SF in men, the risk of MetS increased 1.15-fold (95% CI: 1.10–1.21). In women, a 50 μg/L increase in SF was associated with a 1.50-fold increase in the risk of MetS (95% CI: 1.15–1.94).

#### 3.3.3 Subgroup, sensitivity analyses, and publication bias analysis

Because of the large variability across the study, we sought to assess possible causes of heterogeneity through subgroup analysis. Subgroup analyses were categorized according to sample size, geographic region, and ferritin measurements and corrected for BMI, CRP, HOMA-IR, ALT, and metabolic syndrome criteria.

Nevertheless, significant differences were found in subgroups of ferritin measurements corrected for CPR and BMI. The correlation between ferritin levels and metabolic syndrome was stronger in studies corrected for CRP [OR = 1.90 (95% CI: 1.41–2.56; *I*^2^ = 82.4%)] versus studies not corrected for CRP [OR = 1.63 (95% CI: 1.47–1.81; *I*^2^ = 24.8%)]. In addition, studies not corrected for BMI showed a stronger correlation between serum ferritin levels and metabolic syndrome compared with studies corrected for BMI (OR = 1.93 vs. OR = 1.60). There was no significant difference in the other subgroups, suggesting that SF can increase the risk of MetS (Table 2).

**Table 2:** Subgroup analysis for the association between SF and MetS.

Subgroup	Number of studies	Odds ratio (95% CI)	*I* ^2^	*p*	*p*-value
Ethnicity					
East-Asian	12	1.71 (1.43, 2.04)	76.50%	<0.05	
Non East-Asian	3	1.84 (1.54, 2.20)	30.70%	0.194	0.631
Sample size ≥ 3, 000					
Yes	10	1.77 (1.46, 2.14)	78.60%	<0.05	
No	5	1.71 (1.48, 1.98)	18.70%	0.277	0.469
Ferritin assay					
RIA	5	2.06 (1.52, 2.79)	86.00%	<0.05	
TIA	3	1.55 (1.25, 1.93)	29.80%	0.223	
CLIA	6	1.65 (1.46, 1.86)	10.30%	0.344	
EIA	1	/	/	/	<0.001
Adjusted for BMI					
Yes	10	1.60 (1.41, 1.81)	31.40%	0.100	
No	5	1.93 (1.50, 2.48)	82.50%	<0.05	<0.001
Adjusted for CRP					
Yes	5	1.90 (1.41, 2.56)	82.40%	<0.05	
No	10	1.63 (1.47, 1.81)	24.80%	0.168	<0.001
Adjusted for HOMA-IR					
Yes	3	1.45 (1.21, 1.73)	0.00%	0.405	
No	12	1.80 (1.54, 2.10)	72.60%	<0.05	0.011
Adjusted for ALT					
Yes	4	1.54 (1.28, 1.85)	14.50%	0.319	
No	11	1.80 (1.52, 2.12)	75.80%	<0.05	0.042
Gender					
Men	11	1.96 (1.56, 2.47)	74.80%	<0.05	
Postmenopausal women	4	1.84 (1.42, 2.38)	24.10%	0.267	
Premenopausal women	4	1.64 (1.06, 2.54)	51.40%	0.104	<0.001
Metabolic syndrome criteria					
ATP III	13	1.88 (1.74, 2.03)	72.20%	<0.05	
IDF	2	1.53 (1.32, 1.77)	0.00%	0.589	0.015

**Figure 4:**
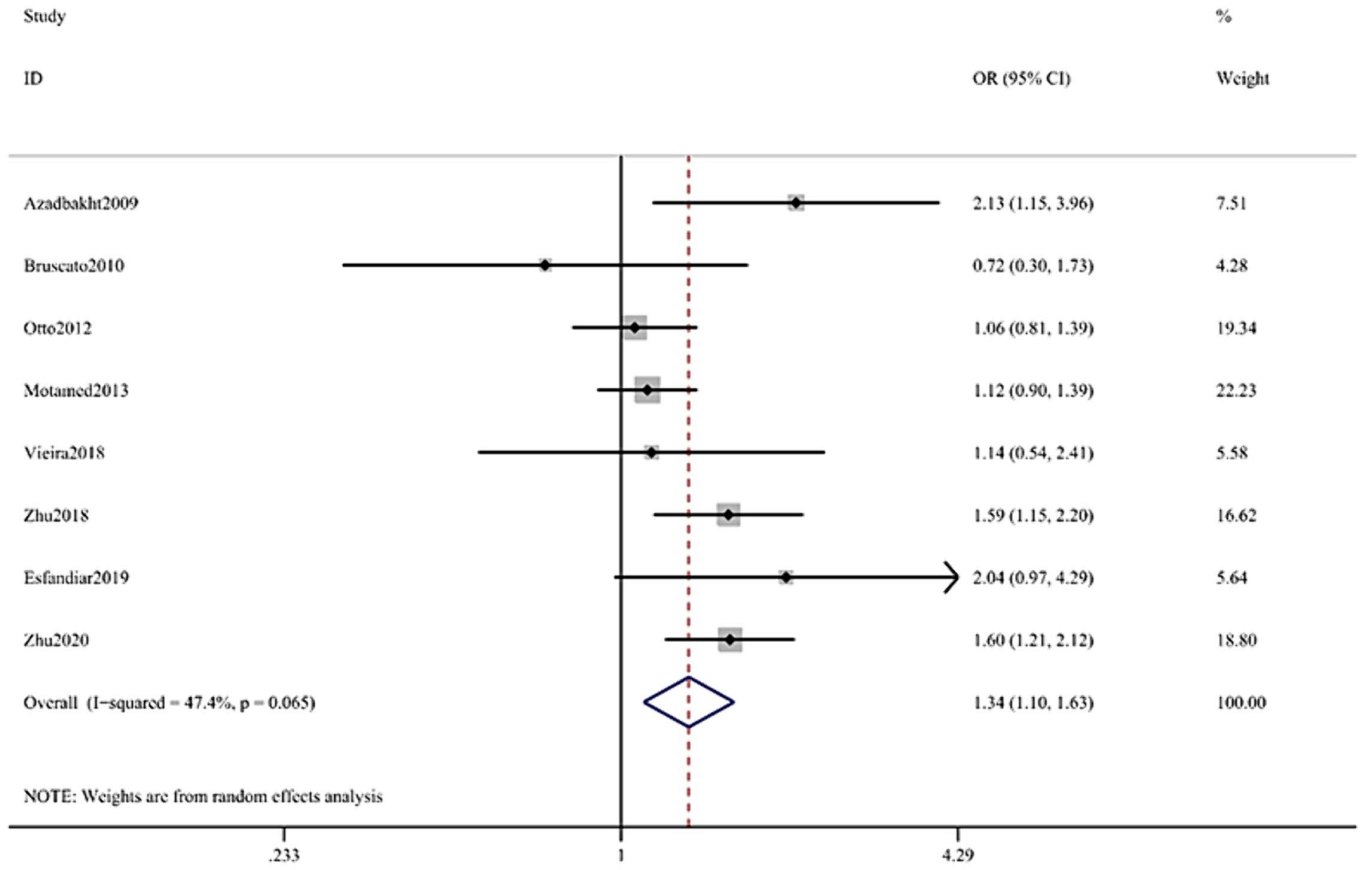
Forest plot of the association between Dietary Iron and MetS in cross-sectional studies. Pooled odds ratios of MetS compared between highest and lowest Dietary Iron categories. MetS, metabolic syndrome.

A sensitivity analysis was conducted to investigate the sources of heterogeneity. By systematically excluding one study at a time and recalculating the pooled odds ratios for the remaining studies, it was observed that removing Li′s study eliminated the heterogeneity (as depicted in Figure 4A). This observation can be attributed to the larger sample size of Li′s study compared to other studies.

**Figure 5:**
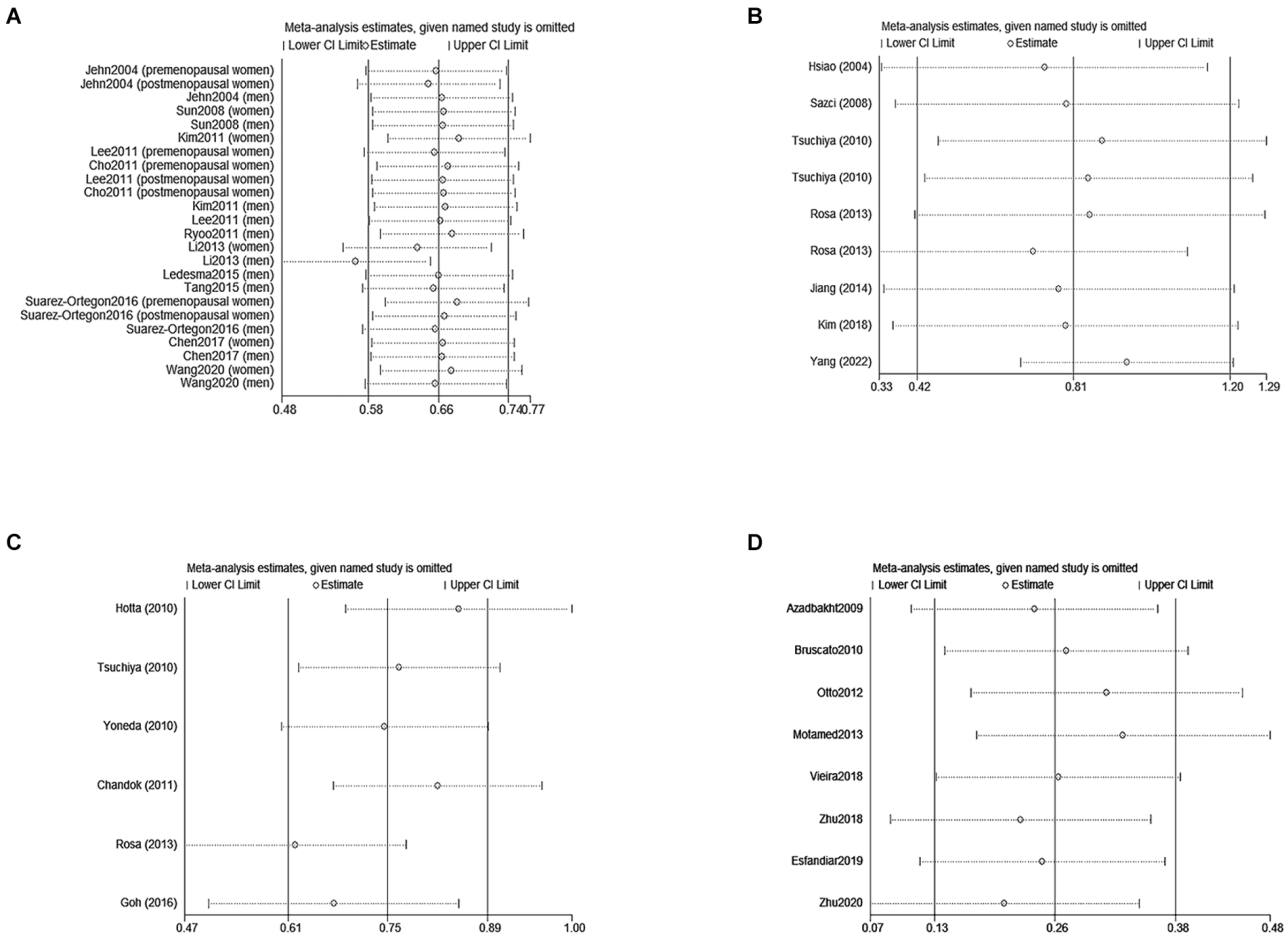
Sensitivity analyses of the association between SF/Dietary Iron and MetS /NAFLD. **(A)** Represents eliminated heterogeneity excluding a study of association between SF and MetS in cross-sectional studies. **(B,C)** Represent eliminated heterogeneity excluding studies of association between SF and NAFLD in cross-sectional studies. **(D)** Represent eliminated heterogeneity excluding studies of association between Dietary and MetS in cross-sectional studies. SF, serum ferritin; NAFLD, non-alcoholic fatty liver disease; MetS, metabolic syndrome.

The log odds ratio’s standard error (SE) for each study was compared to the OR on Egger’s funnel plot (Figure 5A) for visual inspection. Despite slight asymmetry observed in the funnel plot, Egger’s test did not detect any indication of publication bias (*p* = 0.132).

### 3.4 SF and NAFLD incidence

#### 3.4.1 High versus low

**Figure 6:**
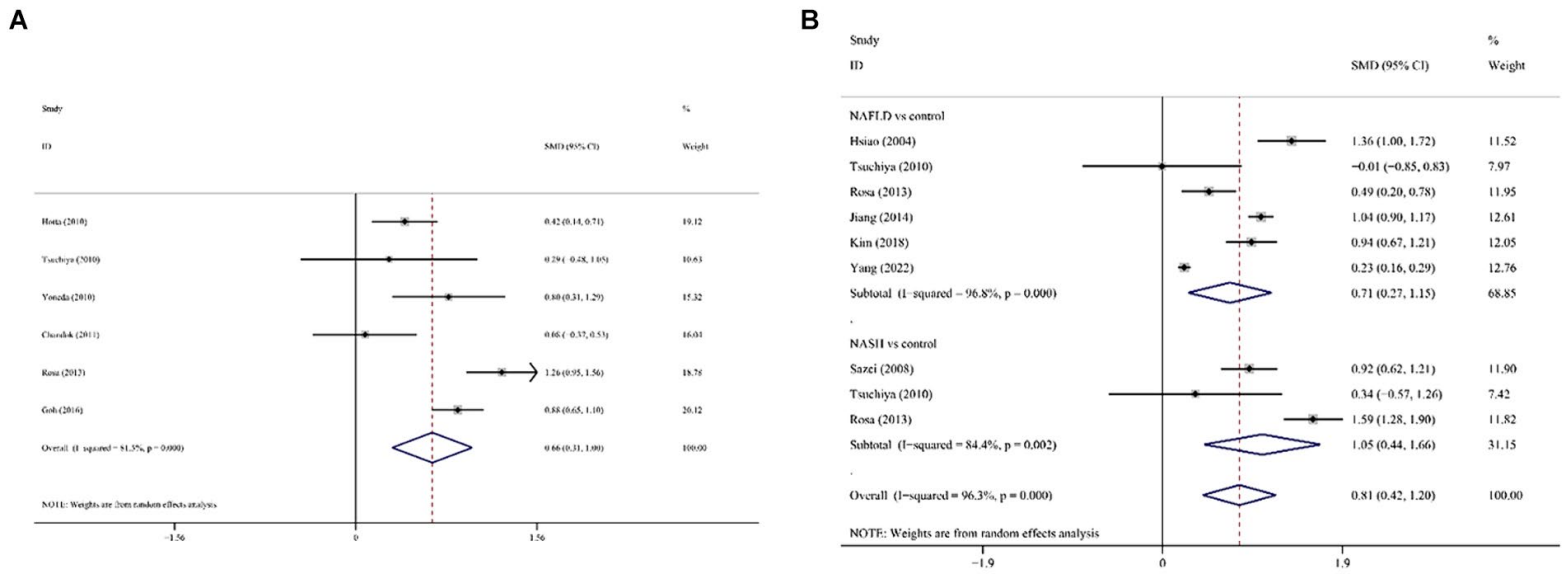
Forest plot of association between SF and NAFLD in cross-sectional studies. **(A)** NAFLD/NASH patients against the control group; **(B)** NASH patients against NAFLD against SF, serum ferritin; NAFLD, non-alcoholic fatty liver disease; NASH, non-alcoholic steatohepatitis.

SF was significantly higher in (1) NAFLD patients compared with the control group; (2) NASH patients compared with the control group and (3) NASH patients compared with NAFLD patients. In all cases of juxtaposition, the differences between studies were severe (*I*^2^ ranged from 81.5 to 96.8%; Figures 6A,B). No meaningful bias was observed in any of the comparisons (*p* > 0.05 for all comparisons; Figure 4).

#### 3.4.2 Dose–response analysis

Combined analysis of baseline SF levels and risk of NAFLD incidence in study subjects of different genders using different models revealed a dose–response relationship between baseline SF levels and risk of NAFLD incidence. There was a non-significant non-linear relationship between SF variants and NAFLD incidence (*p* = 0.2011 Figure 7B). Linear trend test (*p* = 0.041 < 0.05).

**Figure 7:**
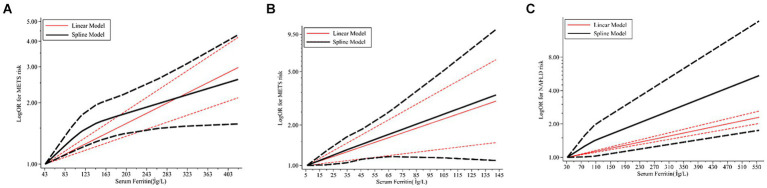
Dose–response relations between SF and risk of MetS/NAFLD in prospective studies. **(A)** Risk between SF and MetS in men estimates from a dose–response meta-analysis. **(B)** Risk between SF and MetS in women estimates from the dose–response meta-analysis. **(C)** Risk between SF and NAFLD estimates from the dose–response meta-analysis. SF, serum ferritin; MetS, metabolic syndrome; NAFLD, non-alcoholic fatty liver disease.

Continuous SMD values for NAFLD incidence in 2 studies were either raw data or extracted by calculation (44, 65). For every 50 μg/L increase in SF, there was a 1.08-fold increase in the risk of NAFLD (95% CI: 1.07–1.10).

#### 3.4.3 Subgroup, sensitivity analyses, and publication bias analysis

Subgroup analyses of the ferritin and NAFLD are shown in Table 3. Stratified analyses were classified by ethnicity, sample size, study design, mean age, NAFLD diagnosis approach/tool, and calculation method.

**Table 3:** Subgroup analysis for the association between SF and NAFLD.

Subgroup	Number of studies	SMD (95% CI)	*I* ^2^	*p*	*p*-value
**(A) NAFLD/NASH patients against the control group**					
*Comparison*					
NAFLD vs. control	6	0.43 (0.34, 0.49)	96.80%	0.000	
NASH vs. control	3	1.19 (0.98, 1.40)	84.40%	0.002	<0.001
*Ethnicity*					
East-Asian	7	0.45 (0.39, 0.50)	96.40%	0.000	
Non East-Asian	2	1.00 (0.79, 1.21)	96.10%	0.000	<0.001
*Sample size ≥ 300*					
Yes	5	0.45 (0.39, 0.50)	97.80%	0.000	
No	4	0.99 (0.79, 1.19)	74.10%	0.000	<0.001
*NAFLD diagnosis approach/tool*					
Liver biopsy	7	0.44 (0.39, 0.50)	96.70%	0.000	
Ultrasonography	2	1.09 (0.87, 1.31)	71.00%	0.063	<0.001
*Study design*					
Case–control	6	0.99 (0.84, 1.14)	86.60%	0.000	
Cross-sectional	3	0.41 (0.35, 0.47)	98.40%	0.000	<0.001
*Calculation method*					
Multivariable logistic regression analysis	8	0.47 (0.41, 0.52)	96.60%	0.000	
Binary logistic regression model	1	/	/	/	0.004
**(B) NASH patients against NAFLD**					
*Ethnicity*					
East-Asian	3	0.97 (0.80, 1.55)	72.10%	0.028	
Non East-Asian	3	0.42 (0.20, 0.63)	56.10%	0.102	<0.001
*Sample size ≥ 300*					
Yes	2	1.01 (0.83, 1.19)	78.00%	0.033	
No	4	0.41 (0.12, 0.61)	82.90%	0.001	0.026
*Study design*					
Case–control	4	0.78 (0.59, 0.97)	82.50%	0.001	
Prospective study	2	0.72 (0.52, 0.92)	89.70%	0.002	0.659
*Calculation method*					
Multivariable logistic regression analysis	5	0.75 (0.61, 0.89)	84.10%	0.000	
Linear regression analysis	1	/	/	/	0.163

However, there were significant differences in the subgroups of ethnicity, sample size, NAFLD diagnosis approach/tool, calculation method, and study design on NAFLD/NASH and control groups. In the NASH and NAFLD groups, there were no statistically significant differences between subgroups for each of the potential confounders (*p* > 0.05 for comparisons between subgroups).

In the sensitivity analysis, the exclusion of individual studies did not significantly change the estimates, and the combined RR ranged from 0.33 to 1.29. Egger’s test was used to assess publication bias. No significant publication bias was observed (Egger’s *p* = 0.104, 0.417, Figures 3B,C).

### 3.5 Dietary iron and MetS incidence

#### 3.5.1 High versus low

The multifactorial adjusted overall RR value showed that dietary iron levels were positively associated with MetS (RR = 1.34, 95% CI: 1.10–1.63; *p* < 0.001) (Figure 4). There was no significant heterogeneity between studies (*p* = 0.065, *I*^2^ = 47.4%).

#### 3.5.2 Subgroup, sensitivity analyses, and publication bias analysis

See Figure 5 for subgroup analyses of dietary iron and MetS. Stratified analyses were categorized according to ethnicity, sample size, adjusted for BMI, study design, mean age, diagnostic criteria of MetS, and exposure assessment. However, differences were statistically significant in racial subgroups corrected for BMI, MetS diagnostic criteria, and exposure assessment. These results were confirmed in the East-Asian (OR = 1.34, 95% CI: 1.21–1.62; *p* = 0.038), no adjusted for BMI (OR = 1.57, 95% CI: 1.23–2.00; *p* = 0.01), the NCEP ATPIII/IDF (OR = 1.59, 95% CI: 1.30–1.93; *p* = 0.002) and 24 h or 3-day recall method (OR = 1.31, 95% CI: 1.07–1.59; *p* = 0.009) in Table 4.

**Table 4:** Subgroup analysis for the association between dietary iron and MetS.

Subgroup	Number of studies	Odds ratio (95% CI)	*I* ^2^	*p*	*p*-value
*Ethnicity*					
East-Asian	5	1.34 (1.21, 1.62)	51.60%	0.083	
Non East-Asian	3	1.04 (0.81, 1.32)	0.00%	0.686	0.038
*Adjusted for BMI*					
Yes	4	1.13 (0.96, 1.33)	0.00%	0.449	
No	4	1.57 (1.23, 2.00)	24.90%	0.262	0.01
*Sample size ≥ 1,000*					
Yes	5	1.34 (1.09, 1.65)	55.40%	0.062	
No	3	1.28 (0.69, 2.38)	53.00%	0.119	0.791
*Study design*					
Cross-sectional	6	1.38 (1.10, 1.73)	47.20%	0.091	
Cohort	2	1.34 (0.72, 2.47)	62.00%	0.105	0.275
*Diagnostic criteria of MetS*					
NCEP ATP III/IDF	7	1.42 (1.14, 1.76)	43.60%	0.100	
Other	1	1.06 (0.81, 1.39)	/	/	0.002
*Exposure assessment*					
FFQ	1	2.04 (0.97, 4.29)	/	/	
24 h and 3 days recall	7	1.31 (1.07, 1.59)	49.20%	0.066	0.009

In the sensitivity analysis, individual studies were excluded that did not significantly change the estimates, and the combined OR was 1.10–1.63. Egger’s test was used to assess publication bias. No significant publication bias was observed (Egger’s *p* = 0.560, Figure 3D).

## 4 Discussion

This meta-analysis aimed to conduct a comprehensive examination of all available data on the association of SF with MetS/NAFLD and the association of dietary iron with MetS and to integrate this information to draw conclusive conclusions about this possible association. There were three primary endpoints of this trial. First, there was a complex association between SF and MetS. We obtained some new results supporting the primary outcome after subgroup analyses, meta-regression analyses, and dose–response analyses. There was a linear dose–response relationship between SF and the prevalence of MetS over a range of values. Each 50 μg/L increase in body SF was associated with a 15% (95% CI: 1.10–1.21) increase in prevalence risk in men and a 50% (95% CI: 1.15–1.94) increase in prevalence risk in women. It was concluded that the risk of metabolic syndrome differed between genders. Second, SF levels were significantly associated with NAFLD. SF levels may increase the incidence of NAFLD. For every 50 μg/L increase in body SF, the incidence of NAFLD increased by approximately 8% (95% CI: 1.07–1.10). Third, the pooled results showed that dietary iron levels were positively associated with MetS.

MetS were proved to be associated with variations on indicators like alanine aminotransferase, uric acid and adiponectin (20, 66, 67). With regard to iron metabolism, it has long been postulated that hyperferritinemia plays a causal role and possesses predictive value in the development of MetS due to its ability to induce inflammation and reactive oxidative stress (ROS). Our prior omics study also found the disturbed iron metabolism had interaction with impaired anti-oxidative capacity (68). Serum ferritin functions as both a signaling molecule and a direct mediator of the immune system (69). Ferritin has been demonstrated to regulate an iron-independent signaling pathway, leading to NF-κB activation and subsequent release of proinflammatory molecules. Chronic inflammation is widely recognized as one of the primary mechanisms underlying MetS (70). Cai and Liu (71) proposed that NF-κB activation serves as the core molecular basis for the pathological process of MetS. Iron, being a transition metal, catalyzes free radical formation which causes oxidative damage in cells and tissues (72). Oxidative stress disrupts the balance between oxidants and antioxidants, resulting in aberrant intracellular signal transmission and gene expression changes. This can contribute to pathological conditions such as insulin resistance (73). Therefore, it has been suggested that ferritin-mediated inflammation and oxidative stress are responsible for linking MetS with Type-2 Diabetes Mellitus (DM) and Cardiovascular Disease (CVD) (74).

To investigate the impact of gender on SF levels and the risk of developing MetS, we observed a slightly lower increased risk of MetS per 1-level increase in SF among men compared to women. Previous studies have highlighted significant differences between women and men regarding insulin action, susceptibility to insulin resistance development, and response to factors influencing insulin sensitivity (75). Females inherently exhibit higher levels of insulin resistance than males, potentially attributed to sex-specific variations in gene expression and metabolic control elements (e.g., signaling pathways, substrate shuttling elements, receptors) (75). Additionally, sex hormones along with environmental and lifestyle factors that either exacerbate or mitigate the genetic predisposition in women may also possess a genetic basis (76). Punnonen et al. (77) investigated the cardiovascular disease (CVD) risk following premenopausal hysterectomy or myomectomy and found that normal uterine function as well as blood loss and iron depletion were essential for maintaining cardiovascular protection in women. However, it is possible that the actual CVD risk in women might not be as high as anticipated. Nevertheless, subgroup analyses were conducted to demonstrate increased heterogeneity based on BMI and CRP levels. After adjusting for BMI, a well-established anthropometric predictor of cardiovascular metabolic disease (CMD) that is positively associated with iron stores, the pooled ferritin-MetS correlations in all evaluated studies were attenuated (78). Fibromodulin is a small leucine-rich proteoglycan that plays a role in the regulation of collagen fibril assembly and has been implicated in various physiological processes, including tissue repair and tumor suppression (79). Adipocytokines have been found to stimulate the synthesis and secretion of fibromodulin hormone, which inhibits intestinal iron absorption and tissue iron release, ultimately leading to iron deficiency. Similarly, obesity-related low-grade inflammation can result in elevated ferritin levels even in the presence of iron deficiency (80). The mechanisms by which insulin resistance affects iron homeostasis explain the association between obesity and excess iron accumulation (81). Future studies should consider incorporating BMI correction when assessing confounders, effect modification, and potential mechanisms. CRP serves as an acute-phase reactant and nonspecific marker of inflammation that has proven utility in predicting future development of cardiovascular disease (82). Recent research has also demonstrated elevated CRP levels in patients with MetS and its predictive value for MetS development (83).

The liver disorders, known as non-alcoholic fatty liver disease (NAFLD), encompass a spectrum ranging from mild hepatic steatosis to non-alcoholic steatohepatitis (NASH) (84). NAFLD is recognized as a metabolic disorder in liver with major impact on hepatic and biliary system (85, 86). Unlike MetS, limited research has been conducted on the longitudinal risk of ferritin about the variability of NAFLD. Our findings indicate that elevated levels of serum ferritin may be linked to the severity of NAFLD, as controls exhibited lower levels compared to patients with NASH or NAFLD, and NAFLD patients had lower levels than NASH patients. Sensitivity analyses and subgroup analyses did not alter these results significantly. Therefore, serum ferritin could potentially serve as a less invasive and more effective biomarker for predicting the progression of NAFLD. Moreover, further insights from age-, sex-, and race-specific longitudinal studies are necessary to fully elucidate the association between serum ferritin and NAFLD.

The pathophysiological mechanisms underlying elevated serum ferritin levels in patients with NAFLD have been investigated by researchers. Hyperferritinemia (HFE) in NAFLD patients is often associated with lipocalin, a marker of insulin resistance and hepatic inflammation (87). Lipocalin-2 (LCN2) is involved in the transport and storage of iron. In NAFLD, disruptions in iron metabolism can lead to iron accumulation in the liver, promoting oxidative stress and hepatocyte injury (88). Alterations in serum iron levels among adults with NAFLD are typically characterized by increased serum ferritin levels and normal transferrin saturation, which is referred to as metabolic abnormal iron overload syndrome (89). Furthermore, serum ferritin levels in NAFLD patients are closely linked to the iron-regulating hormone hepcidin and hepatic iron concentrations (90). In male obese adolescents, elevated serum ferritin levels primarily result from hepatic fat content and inflammation rather than body iron status (91). Elevated serum ferritin levels in NAFLD patients are associated with abnormal markers of iron metabolism or inflammation due partly to *β*-cell dysfunction and insulin resistance (92). In 2001, Manousou et al. (93) found that hyperferritinemia with normal transferrin saturation indicates glucose/lipid metabolism disorders; when combined with various metabolic abnormalities and iron overload, it identifies individuals at risk for NASH. Bugianesi et al. (94) demonstrated that elevated ferritin levels serve as an indicator of severe histological damage rather than iron overload, and the contribution of iron loading and HFE mutations to hepatic fibrosis in most patients with non-alcoholic fatty liver disease (NAFLD) is not significant. In contrast, Angulo et al. (95) found that although serum ferritin levels were associated with more severe hepatic fibrosis, the results of ferritin assays did not provide a satisfactory classification for fibrosis or improve the accuracy of noninvasive scoring when including serum ferritin. Through corrected multivariate logistic regression analysis, they concluded that serum ferritin levels alone exhibited low diagnostic accuracy for detecting the presence or severity of liver fibrosis in NAFLD patients.

In our research, we have identified a positive correlation between dietary iron consumption (both total iron and hemoglobin) and various components of MetS. Our analysis indicates that higher intakes of dietary iron, particularly from non-heme sources, are associated with a modest increase in the risk of developing MetS. This relationship remains significant after adjusting for potential confounders such as age, gender, physical activity levels, and overall dietary patterns. Considering the pivotal role of dietary iron intake in determining body iron stores, it is plausible to propose that higher levels of iron intake may be associated with an increased risk of developing MetS. Humans obtain iron from their diet in two forms: non-heme and heme iron (96). It is widely recognized that the absorption of heme iron remains relatively unaffected by other dietary factors compared to non-heme iron absorption. Several dietary elements, including heme iron, supplemental iron, Vitamin C from food sources, animal protein, copper, and alcohol exhibited significant positive associations with diabetes. Conversely, coffee consumption along with phytate, oxalate, zinc, and calcium intake demonstrated significant negative associations with the occurrence of diabetes (97, 98). Our findings have practical implications for individuals managing MetS daily. For instance, there should be an emphasis on providing comprehensive dietary education specifically tailored for individuals with metabolic syndrome which includes guidance on avoiding excessive intake of dietary sources rich in iron.

The mention of the robustness and applicability of our findings is essential. All literature included in this study met the predefined inclusion criteria. The selected studies collectively demonstrated a high level of quality, establishing a strong basis for evaluating their relevance. SF displayed an approximately linear dose–response correlation, thereby serving as a valuable biomarker for cost-effective prediction and screening of metabolic disorders. Considering the potential causal relationship between hyperferritinemia and metabolic abnormalities, reducing ferritin levels may emerge as a promising therapeutic target in preventing conditions such as metabolic syndrome or NAFLD, which are prevalent risk factors for serious diseases like CVD.

The findings of the meta-analysis were robust; however, certain limitations should be considered in this study. The evaluation primarily relied on cross-sectional studies due to the absence of randomized controlled trials and longitudinal studies. Future research should aim to validate the association between SF-Mets in other ethnicities, as most prospective studies focused on East Asian populations. Combining the results from different studies posed a challenge due to inconsistencies in risk indicators and statistical methods used. To enhance comparability, HR values were converted into OR values before data integration. Additionally, variations in diagnostic criteria for MetS among the included studies may introduce bias when distinguishing patients from non-patients (Supplementary Table S4).

In summary, a meta-analysis of prospective studies has consistently demonstrated a direct relationship between elevated ferritin levels and the incidence of MetS/NAFLD. Moreover, there is a positive correlation between dietary iron intake and MetS. The association between ferritin and MetS may be confounded by BMI and CRP levels. Incorporating ferritin alongside other metabolic and biochemical parameters could improve the accuracy of non-invasive scoring systems for NAFLD. Additionally, targeting iron depletion remains an attractive therapeutic strategy to prevent liver damage progression.

## Data Availability

The original contributions presented in the study are included in the article/Supplementary material, further inquiries can be directed to the corresponding author.
