# Supplementary material for: Dose–response relationship of serum ferritin and dietary iron intake with metabolic syndrome and non-alcoholic fatty liver disease incidence: a systematic review and meta-analysis
Source: Front Nutr. 2024 Oct 1;11:1437681. doi: 10.3389/fnut.2024.1437681 (PMC11476413; doi:10.3389/fnut.2024.1437681)
Supplement: Supplementary file 1 [file Data_Sheet_1.pdf]

Dose-response Relationship of Serum Ferritin and Dietary Iron Intake with Metabolic Syndrome and Non-alcoholic Fatty Liver Disease Incidence: A systematic review and meta-analysis

Lu Yu[1,2,3,4] †, Ting Que[5] † ,Yifeng Zhou[2], Zhengtao Liu[2,3,4,6,7]*

[1] School of Medicine, Zhejiang Chinese Medical University, 310053, Hangzhou, China
[2] Shulan International Medical College, Zhejiang Shuren University, Hangzhou, 310 015, Zhejiang, China
[3] Key Laboratory of Artificial Organs and Computational Medicine in Zhejiang Province, Shulan International Medical College, Zhejiang Shuren University, 310 015, Hangzhou, Zhejiang, China
[4] Shulan (Hangzhou) Hospital, 310 002 Hangzhou, China
[5] Birth Defects Prevention and Control Institute, Maternal and Child Health Hospital of Guangxi Zhuang Autonomous Region, 530 002,Nanning, China
[6] NHC Key Laboratory of Combined Multi-organ Transplantation, Key Laboratory of the diagnosis and treatment of organ Transplantation, CAMS, First Affiliated Hospital, School of Medicine, Zhejiang University, 310 003, Hangzhou, China
[7] Key Laboratory of Organ Transplantation, Zhejiang Province, First Affiliated Hospital, School of Medicine, Zhejiang University, 310 003, Hangzhou, China

† These authors have contributed equally to this work.
*** Correspondence:** Zhengtao Liu, Shulan International Medical College, Zhejiang Shuren University, Hangzhou, 310015, Zhejiang, China
Email: liuzhengtao@zjsru.edu.cn

frontiers

PRISMA 2020 flow diagram

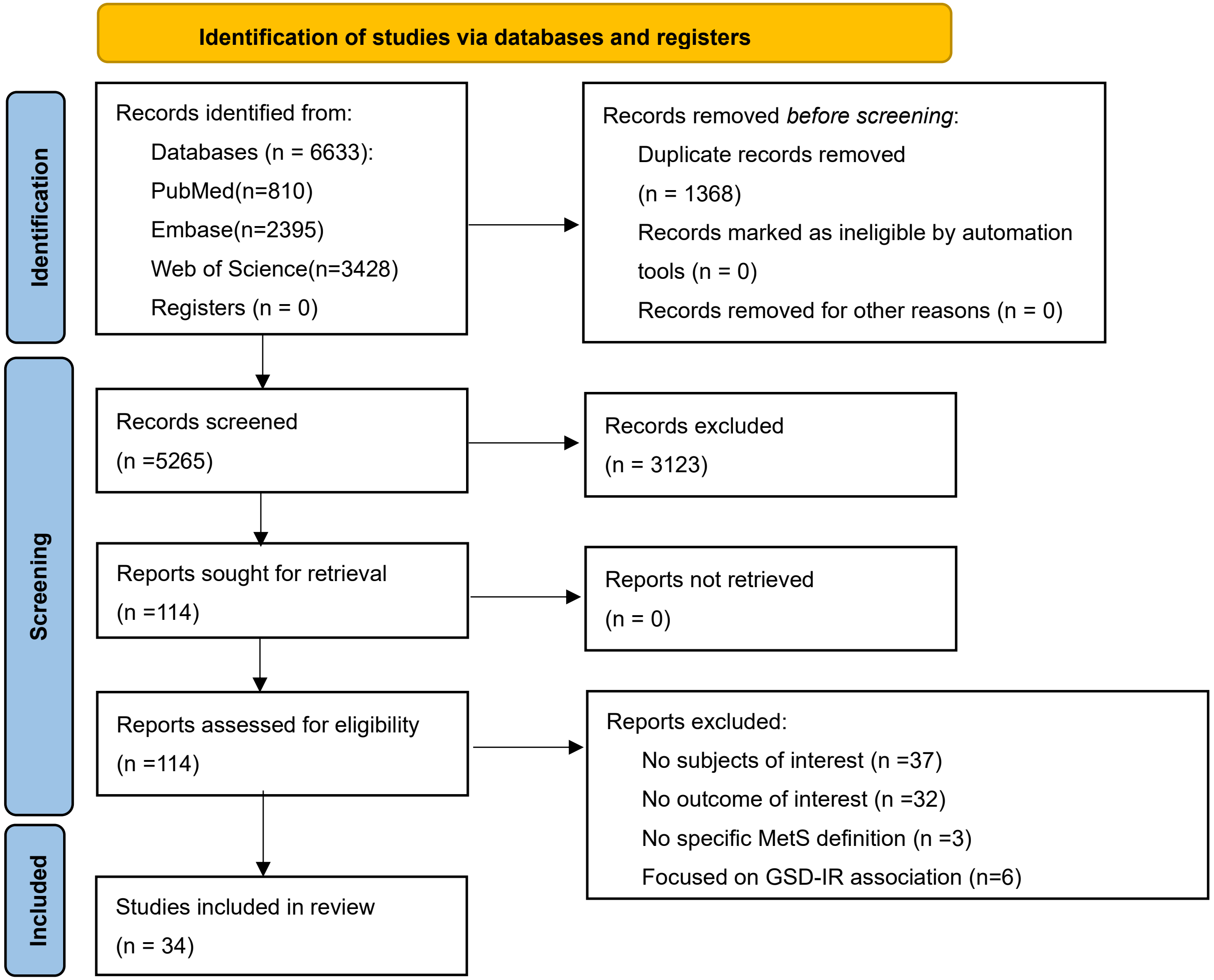

From: Page MJ, McKenzie JE, Bossuyt PM, Boutron I, Hoffmann TC, Mulrow CD, et al. The PRISMA 2020 statement: an updated guideline for reporting systematic reviews. BMJ 2021;372:n71. doi: 10.1136/bmj.n71

For more information, visit: http://www.prisma-statement.o

PRISMA 2020 for Abstracts Checklist

Section and Topic	Item #	Checklist item	Reported (Yes/No)
**TITLE**			
Title	1	Identify the report as a systematic review.	Yes
**BACKGROUND**			
Objectives	2	Provide an explicit statement of the main objective(s) or question(s) the review addresses.	Yes
**METHODS**			
Eligibility criteria	3	Specify the inclusion and exclusion criteria for the review.	Yes
Information sources	4	Specify the information sources (e.g. databases, registers) used to identify studies and the date when each was last searched.	Yes
Risk of bias	5	Specify the methods used to assess risk of bias in the included studies.	Yes
Synthesis of results	6	Specify the methods used to present and synthesise results.	Yes
**RESULTS**			
Included studies	7	Give the total number of included studies and participants and summarise relevant characteristics of studies.	Yes
Synthesis of results	8	Present results for main outcomes, preferably indicating the number of included studies and participants for each. If meta-analysis was done, report the summary estimate and confidence/credible interval. If comparing groups, indicate the direction of the effect (i.e. which group is favoured).	Yes
**DISCUSSION**			
Limitations of evidence	9	Provide a brief summary of the limitations of the evidence included in the review (e.g. study risk of bias, inconsistency and imprecision).	Yes
Interpretation	10	Provide a general interpretation of the results and important implications.	Yes
**OTHER**			
Funding	11	Specify the primary source of funding for the review.	Yes
Registration	12	Provide the register name and registration number.	No

From: Page MJ, McKenzie JE, Bossuyt PM, Boutron I, Hoffmann TC, Mulrow CD, et al. The PRISMA 2020 statement: an updated guideline for reporting systematic reviews. BMJ 2021;372:n71. doi: 10.1136/bmj.n71

For more information, visit: http://www.prisma-statement.org/

PRISMA 2020 Checklist

Section and Topic	Item #	Checklist item	Location where item is reported
**TITLE**			
Title	1	Identify the report as a systematic review.	1
**ABSTRACT**			
Abstract	2	See the PRISMA 2020 for Abstracts checklist.	2
**INTRODUCTION**			
Rationale	3	Describe the rationale for the review in the context of existing knowledge.	3
Objectives	4	Provide an explicit statement of the objective(s) or question(s) the review addresses.	3-4
**METHODS**			
Eligibility criteria	5	Specify the inclusion and exclusion criteria for the review and how studies were grouped for the syntheses.	3
Information sources	6	Specify all databases, registers, websites, organisations, reference lists and other sources searched or consulted to identify studies. Specify the date when each source was last searched or consulted.	3
Search strategy	7	Present the full search strategies for all databases, registers and websites, including any filters and limits used.	3
Selection process	8	Specify the methods used to decide whether a study met the inclusion criteria of the review, including how many reviewers screened each record and each report retrieved, whether they worked independently, and if applicable, details of automation tools used in the process.	3
Data collection process	9	Specify the methods used to collect data from reports, including how many reviewers collected data from each report, whether they worked independently, any processes for obtaining or confirming data from study investigators, and if applicable, details of automation tools used in the process.	3
Data items	10a	List and define all outcomes for which data were sought. Specify whether all results that were compatible with each outcome domain in each study were sought (e.g. for all measures, time points, analyses), and if not, the methods used to decide which results to collect.	3
	10b	List and define all other variables for which data were sought (e.g. participant and intervention characteristics, funding sources). Describe any assumptions made about any missing or unclear information.	3
Study risk of bias assessment	11	Specify the methods used to assess risk of bias in the included studies, including details of the tool(s) used, how many reviewers assessed each study and whether they worked independently, and if applicable, details of automation tools used in the process.	3
Effect measures	12	Specify for each outcome the effect measure(s) (e.g. risk ratio, mean difference) used in the synthesis or presentation of results.	3

Section and Topic	Item #	Checklist item	Location where item is reported
Synthesis methods	13a	Describe the processes used to decide which studies were eligible for each synthesis (e.g. tabulating the study intervention characteristics and comparing against the planned groups for each synthesis (item #5)).	3
	13b	Describe any methods required to prepare the data for presentation or synthesis, such as handling of missing summary statistics, or data conversions.	3
	13c	Describe any methods used to tabulate or visually display results of individual studies and syntheses.	3
	13d	Describe any methods used to synthesize results and provide a rationale for the choice(s). If meta-analysis was performed, describe the model(s), method(s) to identify the presence and extent of statistical heterogeneity, and software package(s) used.	3
	13e	Describe any methods used to explore possible causes of heterogeneity among study results (e.g. subgroup analysis, meta-regression).	3
	13f	Describe any sensitivity analyses conducted to assess robustness of the synthesized results.	3
Reporting bias assessment	14	Describe any methods used to assess risk of bias due to missing results in a synthesis (arising from reporting biases).	3
Certainty assessment	15	Describe any methods used to assess certainty (or confidence) in the body of evidence for an outcome.	3
**RESULTS**			
Study selection	16a	Describe the results of the search and selection process, from the number of records identified in the search to the number of studies included in the review, ideally using a flow diagram.	3
	16b	Cite studies that might appear to meet the inclusion criteria, but which were excluded, and explain why they were excluded.	3
Study characteristics	17	Cite each included study and present its characteristics.	3
Risk of bias in studies	18	Present assessments of risk of bias for each included study.	3
Results of individual studies	19	For all outcomes, present, for each study: (a) summary statistics for each group (where appropriate) and (b) an effect estimate and its precision (e.g. confidence/credible interval), ideally using structured tables or plots.	4
Results of syntheses	20a	For each synthesis, briefly summarise the characteristics and risk of bias among contributing studies.	5-7
	20b	Present results of all statistical syntheses conducted. If meta-analysis was done, present for each the summary estimate and its precision (e.g. confidence/credible interval) and measures of statistical heterogeneity. If comparing groups, describe the direction of the effect.	5-7

Section and Topic	Item #	Checklist item	Location where item is reported
	20c	Present results of all investigations of possible causes of heterogeneity among study results.	5-7
	20d	Present results of all sensitivity analyses conducted to assess the robustness of the synthesized results.	5-7
Reporting biases	21	Present assessments of risk of bias due to missing results (arising from reporting biases) for each synthesis assessed.	5-7
Certainty of evidence	22	Present assessments of certainty (or confidence) in the body of evidence for each outcome assessed.	5-7
**DISCUSSION**			
Discussion	23a	Provide a general interpretation of the results in the context of other evidence.	7-9
	23b	Discuss any limitations of the evidence included in the review.	8
	23c	Discuss any limitations of the review processes used.	8
	23d	Discuss implications of the results for practice, policy, and future research.	8
**OTHER INFORMATION**			
Registration and protocol	24a	Provide registration information for the review, including register name and registration number, or state that the review was not registered.	3
	24b	Indicate where the review protocol can be accessed, or state that a protocol was not prepared.	3
	24c	Describe and explain any amendments to information provided at registration or in the protocol.	3
Support	25	Describe sources of financial or non-financial support for the review, and the role of the funders or sponsors in the review.	10
Competing interests	26	Declare any competing interests of review authors.	10

From: Page MJ, McKenzie JE, Bossuyt PM, Boutron I, Hoffmann TC, Mulrow CD, et al. The PRISMA 2020 statement: an updated guideline for reporting systematic reviews. BMJ 2021;372:n71. doi: 10.1136/bmj.n71

For more information, visit: http://www.prisma-statement.org/

FigureS1. study flow diagram

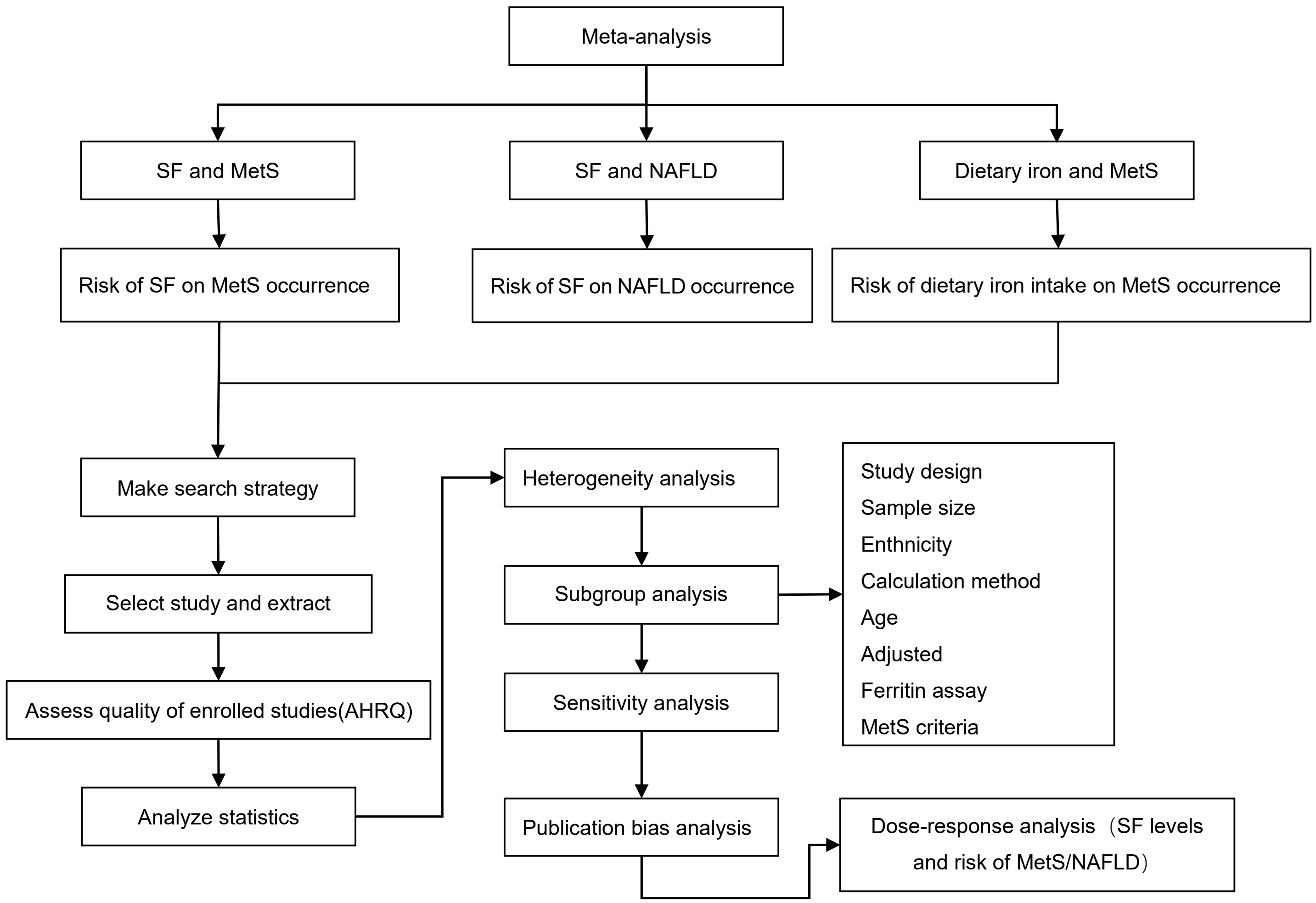

Table S1 Search strategy for literatures about the association of serum ferritin / iron with metabolic syndrome and non-alcoholic fatty liver disease

Pubmed	Search strings	Results
1	metabolic syndrome [MeSH Terms]	38571
2	syndrome x [Text Word]	2235
3	insulin resistance syndrome [Text Word]	1749
4	MetS [Text Word]	15360
5	1 OR 2 OR 3 OR 4	47977
6	non alcoholic fatty liver disease [MeSH Terms]	24205
7	nonalcoholic steatohepatitis [Text Word]	8005
8	NAFLD [Text Word]	25533
9	NASH [Text Word]	13570
10	6 OR 7 OR 8 OR 9	39285
11	serum ferritin [Text Word]	11187
12	SF [Text Word]	62269
13	11 OR 12	72589
14	iron [Text Word]	264776
15	Fe [Text Word]	147171
16	14 OR 15	356331
17	5 AND 13	291
18	5 AND 16	310
19	10 AND 13	209

Embase		
1	'metabolic syndrome x'/exp OR 'metabolic syndrome x'	101862
2	'syndrome x'	105338
3	'insulin resistance syndrome'	2207
4	'mets'	26061
5	1 OR 2 OR 3 OR 4	116167
6	'nonalcoholic fatty liver'/exp OR 'nonalcoholic fatty liver'	73245
7	'nonalcoholic steatohepatitis'	20220

8	'nafld'	44233
9	'nash'	41330
10	6 OR 7 OR 8 OR 9	97467
11	'ferritin blood level'/exp OR 'ferritin blood level'	27905
12	'serum ferritin'	17624
13	11 OR 12	32778
14	'iron'/exp OR 'iron'	402786
15	'Fe'	205548
16	14 OR 15	514884
17	5 AND 13	390
18	5 AND 16	1381
19	10 AND 13	624

Web of Science		
1	Topic=('metabolic syndrome ')	145057
2	Topic=('syndrome x')	54773
3	Topic=('insulin resistance syndrome')	55121
4	Topic=('MetS')	15336
5	1 OR 2 OR 3 OR 4	206021
6	Topic=('non alcoholic fatty liver disease')	21496
7	Topic=('nonalcoholic steatohepatitis')	18129
8	Topic=('NAFLD')	28271
9	Topic=('NASH')	49183
10	6 OR 7 OR 8 OR 9	84060
11	Topic=('serum ferritin')	17528
12	Topic=('SF')	85656
13	11 OR 12	102405
14	Topic=('iron')	693194
15	Topic=('Fe')	602969
16	14 OR 15	1117201
17	5 AND 13	1174
18	5 AND 16	1871

19	10 AND 13	383

Table S2 Check List for Quality Assessment and Scoring of Nonrandomized Studies

Check List

Selection
1. Define the source of information (survey, record review)
2. List inclusion and exclusion criteria for exposed and unexposed subjects (cases and controls) or refer to previous publications
3. Indicate time period used for identifying patients
4. Indicate whether or not subjects were consecutive if not population-based
5. Indicate if evaluators of subjective components of study were masked to other aspects of the status of the participants
6. Describe any assessments undertaken for quality assurance purposes (e.g., test/retest of primary outcome measurements)
7. Explain any patient exclusions from analysis
8. Describe how confounding was assessed and/or controlled
9. If applicable, explain how missing data were handled in the analysis
10. Summarize patient response rates and completeness of data collection
11. Clarify what follow-up, if any, was expected and the percentage of patients for which incomplete data or follow-up was obtained

Table S3 Quality assessment of the studies included in the meta-analysis by AHRQ

AHRQ scale	Wang et al. 2020	Li et al. 2013	Chen et al. 2017	Jehn et al. 2004	Suárez-Ortegón et al. 2016	Shim et al. 2017	Kim et al. 2011
**1.Define the source of information (survey, record review)**	1	1	1	1	1	1	1
**2.List inclusion and exclusion criteria for exposed and unexposed subjects (cases and controls) or refer to previous publications**	1	1	1	1	1	1	1
**3.Indicate time period used for identifying patients**	1	1	1	1	1	1	1
**4.Indicate whether or not subjects were consecutive if not population-based**	0	1	1	0	1	1	1
**5.Indicate if evaluators of subjective components of study were masked to other aspects of the status of the participants**	1	1	1	1	1	1	1
**6.Describe any assessments undertaken for quality assurance purposes**	1	0	1	1	0	1	0
**7.Explain any patient exclusions from analysis**	0	0	1	0	1	0	1
**8.Describe how confounding was assessed and/or controlled**							
**9.If applicable, explain how missing data were handled in the analysis**	0	1	0	0	1	0	1
**10.Summarize patient response rates and completeness of data collection**	1	1	1	1	1	1	1
**11.Clarify what follow-up, if any, was expected and the percentage of patients for which incomplete data or follow-up was obtained**	1	0	0	1	0	0	0
**Total scores (maximum 11)**	8	8	9	8	9	8	9

Continued

AHRQ scale	Lee et al. 2011	Kang et al. 2012	Chang et al. 2013	Cho et al. 2011	Ryoo et al. 2011	Ledesma et al. 2015	Tang et al. 2015
**1. Define the source of information (survey, record review)**	1	1	1	1	1	1	1
**2.List inclusion and exclusion criteria for exposed and unexposed subjects (cases and controls) or refer to previous publications**	0	0	0	1	1	0	1
**3.Indicate time period used for identifying patients**	1	1	1	1	1	1	1
**4.Indicate whether or not subjects were consecutive if not population-based**	1	1	1	1	1	1	1
**5.Indicate if evaluators of subjective components of study were masked to other aspects of the status of the participants**	0	0	0	0	1	1	1
**6.Describe any assessments undertaken for quality assurance purposes**	1	1	1	1	1	1	0
**7.Explain any patient exclusions from analysis**	1	1	0	0	0	0	1
**8.Describe how confounding was assessed and/or controlled**	0	0	1	1	1	1	1
**9.If applicable, explain how missing data were handled in the analysis**	1	1	1	0	0	0	1
**10.Summarize patient response rates and completeness of data collection**							
**11.Clarify what follow-up, if any, was expected and the percentage of patients for which incomplete data or follow-up was obtained**	1	1	1	1	1	1	1
**Total scores (maximum 9)**	8	8	8	8	9	8	10

Continued

AHRQ scale	Chandok et al. 2010	Hotta et al. 2010	Hsiao et al. 2004	Goh et al. 2016	Rosa et al. 2013	Sazci et al. 2008	Jiang et al. 2014
**1.Define the source of information (survey, record review)**	1	1	1	1	1	1	1
**2.List inclusion and exclusion criteria for exposed and unexposed subjects (cases and controls) or refer to previous publications**	1	0	0	1	1	0	1
**3.Indicate time period used for identifying patients**	1	1	1	1	1	1	1
**4.Indicate whether or not subjects were consecutive if not population-based**	0	1	1	0	0	1	0
**5.Indicate if evaluators of subjective components of study were masked to other aspects of the status of the participants**	1	1	1	1	1	1	1
**6.Describe any assessments undertaken for quality assurance purposes**	0	0	1	1	0	1	0
**7.Explain any patient exclusions from analysis**	1	1	1	1	1	1	1
**8.Describe how confounding was assessed and/or controlled**	1	1	1	1	1	0	1
**9.If applicable, explain how missing data were handled in the analysis**	1	1	1	1	1	1	1
**10.Summarize patient response rates and completeness of data collection**	1	1	1	1	1	1	1
**11.Clarify what follow-up, if any, was expected and the percentage of patients for which incomplete data or follow-up was obtained**	0	0	0	0	0	1	1
**Total scores (maximum 9)**	8	8	9	9	8	9	9

Continued

AHRQ scale	Yoneda et al. 2010	Kim et al. 2018	Yang et al. 2022	Tsuchiya et al. 2010	Azadbakh et al. 2009	Bruscato et al. 2010	Otto et al. 2012
**1. Define the source of information (survey, record review)**	1	1	1	1	1	1	1
**2.List inclusion and exclusion criteria for exposed and unexposed subjects (cases and controls) or refer to previous publications**	1	1	1	1	0	1	0
**3.Indicate time period used for identifying patients**	1	0	1	1	0	0	1
**4.Indicate whether or not subjects were consecutive if not population-based**	0	0	0	0	1	1	1
**5.Indicate if evaluators of subjective components of study were masked to other aspects of the status of the participants**	1	1	1	1	1	1	1
**6.Describe any assessments undertaken for quality assurance purposes**	0	0	0	1	1	1	1
**7.Explain any patient exclusions from analysis**	1	1	1	1	1	1	1
**8.Describe how confounding was assessed and/or controlled**	1	1	1	1	1	1	1
**9.If applicable, explain how missing data were handled in the analysis**	1	1	1	1	1	1	0
**10.Summarize patient response rates and completeness of data collection**	0	0	0	0	0	0	1
**11.Clarify what follow-up, if any, was expected and the percentage of patients for which incomplete data or follow-up was obtained**	1	1	1	0	1	0	1
**Total scores (maximum 9)**	8	7	8	8	8	8	9

Continued

AHRQ scale	Motamed et al. 2013	Zhu et al. 2018	Vieira et al. 2018	Esfandiar et al. 2019	Zhu et al. 2020
**1.Define the source of information (survey, record review)**	**1**	**1**	**1**	**1**	**1**
**2.List inclusion and exclusion criteria for exposed and unexposed subjects (cases and controls) or refer to previous publications**	1	1	1	1	1
**3.Indicate time period used for identifying patients**	1	1	1	1	1
**4.Indicate whether or not subjects were consecutive if not population-based**	0	0	0	0	0
**5.Indicate if evaluators of subjective components of study were masked to other aspects of the status of the participants**	1	1	1	1	0
**6.Describe any assessments undertaken for quality assurance purposes**	**1**	1	0	1	1
**7.Explain any patient exclusions from analysis**	**1**	1	1	1	1
**8.Describe how confounding was assessed and/or controlled**	**0**	0	0	0	1
**9.If applicable, explain how missing data were handled in the analysis**	**1**	1	1	1	1
**10.Summarize patient response rates and completeness of data collection**	1	1	1	1	1
**11.Clarify what follow-up, if any, was expected and the percentage of patients for which incomplete data or follow-up was obtained**	1	1	1	1	1
**Total scores (maximum 9)**	9	9	8	9	9

Table S4 Definition of MetS and its Related Components in Enrolled Studies

Author, publication(ref)	Obesity	Hypertriglyceridemia	low HDL-C	Hyperglycemia	Hypertension	Diagnostic criteria	MetS definition
Chang et al.2013	WC >90cm for men<br>WC>80cm for women	TG >150mg/dL	HDL-C<40mg/dL for men;<br>HDL-C<50mg/dL for women;<br>or medication for improving HDL-C	FBG≥100mg/dL or medication for anti-hyperglycemia	SBP≥130mmHg and/or DBP≥85mmHg or the medication of anti-hypertension	Three of the six criteria were grounds for definition	NCEP-ATP-III on the Asia-Pacific criteria
Cho et al.2011	WC≥85cm for women	TG >150mg/dL	HDL-C<50mg/dL for women	FBG≥100mg/dL or medication for anti-hyperglycemia	SBP≥130mmHg and/or DBP≥85mmHg or the medication of anti-hypertension	Three of the five criteria were grounds for definition	NCEP-ATP-III
Li et al.2013	WC >90cm for men<br>WC>80cm for women	TG ≥1.7 mmol/L(≥ 150 mg/dl)	HDL-C<40mg/dL for men;<br>HDL-C<50mg/dL for women;	FBG≥6.1 mmol/L (≥ 110mg/dL)	SBP≥130mmHg and/or DBP≥85mmHg	Three of the five criteria were grounds for definition	NCEP-ATPIII for Asian-Americans
Kim et al.2011	WC >90cm for men<br>WC>80cm for women	TG ≥1.7 mmol/L	HDL-C< 1.03 mmol/L for men;<br>HDL-C< 1.29 mmol/L for women;	FBG≥5.6 mmol/L or medication for anti-hyperglycemia	SBP≥130mmHg and/or DBP≥85mmHg or the medication of anti-hypertension	Three of the five criteria were grounds for definition	NCEP-ATP-III
Sun et al.2008	WC >90cm for men<br>WC>80cm for women	TG ≥1.7 mmol/L	HDL-C< 1.03 mmol/L for men;<br>HDL-C< 1.30 mmol/L for women;	FBG≥5.6 mmol/L or medication for anti-hyperglycemia	SBP≥130mmHg and/or DBP≥85mmHg or the medication of anti-hypertension	Three of the five criteria were grounds for definition	NCEP-ATPIII for Asian-Americans
Ledesma et al.20115	WC >102cm	TG ≥1.7 mmol/L(≥ 150 mg/dl) or drug treatment for elevated	HDL-C< 1.03 mmol/L or drug treatment for depressed HDL-	FBG≥5.6 mmol/L or medication for anti-hyperglycemia	SBP≥130mmHg and/or DBP≥85mmHg or	Three of the five criteria were grounds for	IDF criteria

		triglycerides	cholesterol concentration		the medication of anti-hypertension	definition	
Lee et al.2011	WC >90cm for men WC>85cm for women	TG >150mg/dL	HDL-C<40mg/dL for men; HDL-C<50mg/dL for women;	FBG≥100 mg/dL or medication for anti-hyperglycemia	SBP≥130mmHg and/or DBP≥85mmHg or the medication of anti-hypertension	Three of the five criteria were grounds for definition	NCEP-ATPIII
Chen et al.2016	WC >90cm for men WC>80cm for women	TG≥1.7 mmol/L	HDL-C < 1.03 mmol/L in men or HDL-C < 1.29 mmol/L in women	FBG≥5.6 mmol/L or medication for anti-hyperglycemia	SBP≥130mmHg and/or DBP≥85mmHg	Central obesity plus any other two abnormalities	IDF criteria
JEHN et al.2004	WC >102cm for men WC>88cm for women	TG≥1.695mmol/L	HDL-C < 1.036 mmol/L in men or HDL-C < 1.295 mmol/L in women	FBG≥6.105 mmol/L or medication for anti-hyperglycemia	SBP≥130mmHg and/or DBP≥85mmHg or the medication of anti-hypertension	Three of the five criteria were grounds for definition	NCEP-ATPIII
Suárez-Ortegón et al.2016	WC >94cm for men WC>80cm for women	TG≥1.7 mmol/L	HDL-C < 1.0 mmol/L in men or HDL-C < 1.3 mmol/L in women	FBG≥5.6 mmol/L or medication for anti-hyperglycemia	SBP≥130mmHg and/or DBP≥85mmHg or the medication of anti-hypertension	Three of the five criteria were grounds for definition	international consensus
Tang et al.2015	WC ≥90cm	TG≥1.7 mmol/L	HDL-C < 1.03 mmol/L	FBG≥5.6 mmol/L or medication for anti-hyperglycemia	SBP≥130mmHg and/or DBP≥85mmHg or the medication of anti-hypertension	Three of the five criteria were grounds for definition	NCEP-ATPIII for Asian-Americans
Ryoo et al.2011	BMI ≥ 25 kg/m²	TG >150mg/dL	HDL-C<40mg/dL for men;	FBG≥110mg/dL	SBP≥130mmHg and/or DBP≥85mmHg or the medication of anti-hypertension	Three of the five criteria were grounds for definition	NCEP-ATPIII

Wang et al.2020	WC >90cm for men WC>85cm for women	TG≥1.7 mmol/L or specific treatment for this lipid abnormality	HDL-C ＜1.0 mmol/L in men or HDL-C ＜ 1.3 mmol/L in women or specific treatment	FBG≥5.6 mmol/L or medication for anti-hyperglycemia	SBP≥130mmHg and/or DBP≥85mmHg or the medication of anti-hypertension	Three of the five criteria were grounds for definition	Joint Interim criteria
Shim et al.2017	WC >90cm for men WC>80cm for women	TG >150mg/dL	HDL-C<40mg/dL for men; HDL-C<50mg/dL for women;	FBG≥110mg/dL or medication for anti-hyperglycemia	SBP≥130mmHg and/or DBP≥85mmHg or the medication of anti-hypertension	Central obesity plus any other two abnormalities	IDF criteria for Asia
Bruscato et al.2010	WC>80cm	TG >150mg/dL	HDL-C<50mg/dL for women;	FBG≥100mg/dL	SBP≥130mmHg and/or DBP≥85mmHg or the medication of anti-hypertension	Central obesity plus any other two abnormalities	IDF criteria
Otto et al.2012	WC >102cm for men WC>88cm for women	TG ≥1.7 mmol/L or treatment for elevated TG	HDL-C ＜ 1.03 mmol/L in men or HDL-C＜1.29 mmol/L in women	FBG≥5.6 mmol/L or medication for anti-hyperglycemia	SBP≥130mmHg and/or DBP≥85mmHg or the medication of anti-hypertension	Three of the five criteria were grounds for definition	AHA/NHLBI criteria
Azadbakht et al.2009	WC>88cm	TG ≥1.65mmol/L	HDL-C＜1.29 mmol/L	FBG≥6.05mmol/L	SBP≥130mmHg and/or DBP≥85mmHg	Three of the five criteria were grounds for definition	NCEP-ATPIII
Motamed et al.2013	WC >94cm for men WC>80cm for women	TG >150mg/dL	HDL-C<40mg/dL for men; HDL-C<50mg/dL for women;	FBG≥110mg/dL	SBP≥130mmHg and/or DBP≥85mmHg	Three of the five criteria were grounds for definition	IDF criteria
Vieira et al.2018	WC >90cm for men WC>80cm for women	TG >150mg/dL or treatment for elevated TG	HDL-C<40mg/dL for men; HDL-C<50mg/dL for women;	FBG≥100mg/dL or medication for anti-hyperglycemia	SBP≥130mmHg and/or DBP≥85mmHg or the medication of anti-hypertension	Three of the five criteria were grounds for definition	NCEP-ATPIII

Zhu et al.2018	WC >90cm for men<br>WC>80cm for women	TG >150mg/dL or treatment for elevated TG	HDL-C<40mg/dL for men;<br>HDL-C<50mg/dL for women; or on drug treatment for reduced HDL‐C	FBG≥100mg/dL or medication for anti-hyperglycemia	SBP≥130mmHg and/or DBP≥85mmHg or the medication of anti-hypertension	Three of the five criteria were grounds for definition	NCEP-ATP III for Asian
Zhu et al.2020	WC >90cm for men<br>WC>80cm for women	TG >150mg/dL or treatment for elevated TG	HDL-C<40mg/dL for men;<br>HDL-C<50mg/dL for women; or on drug treatment for reduced HDL‐C	FBG≥100mg/dL or medication for anti-hyperglycemia	SBP≥130mmHg and/or DBP≥85mmHg or the medication of anti-hypertension	Three of the five criteria were grounds for definition	NCEP-ATP III for Asian
Esfandiar et al.2019	WC ≥ 95 cm in men and women	TG≥ 1.70 mmol/l (≥ 150 mg/dl) or drug treatment.	HDL-C < 1.30 mmol/l (< 50 mg/dl) in women, and < 1.04 mmol/l (< 40 mg/dl) in men or receiving drug treatment;	FBS ≥ 6.11 mmol/l (≥ 110 mg/dl) or drug treatment for hyperglycemia	SBP≥130mmHg and/or DBP≥85mmHg or the medication of anti-hypertension	Three of the five criteria were grounds for definition	NCEP-ATP III for Iranian

Abbreviations: BMI, body mass index; FBG, fasting blood glucose; DBP , diastolic blood pressure; HDL-C, high density lipoprotein cholesterol; IDF , International Diabetes Federation; MetS, metabolic syndrome; NCEP-ATP-III, National Cholesterol Education Program Adult Treatment Panel III; SBP , systolic blood pressure; TG, triglyceride; WC, waist circumference.
